# Charge-Shifting Copolymers of 2‑(*N*,*N*‑Dimethylamino)Ethyl Acrylate and 2‑Hydroxyethyl Acrylate via RAFT Polymerization: Balancing the Charge Content and Biological Response

**DOI:** 10.1021/acsapm.5c04108

**Published:** 2026-03-01

**Authors:** Radoslava Sivkova, Monika Matiyani, Gabriela S. García-Briones, Rafal Konefal, Volodymyr Lobaz, Lenka Kotrchová, Elena Filová, Natália Podhorská, Libor Kostka, Dana Kubies

**Affiliations:** † Institute of Macromolecular Chemistry, 86879 Czech Academy of Sciences, Heyrovsky nam. 2, Prague 162 00 Prague 6, Czech Republic; ‡ NanoBioMedical Centre, Adam Mickiewicz University, Wszechnicy Piastowskiej 3, Poznań 61-614, Poland; § Laboratory of Biomaterials and Tissue Engineering, Institute of Physiology of the Czech Academy of Sciences, Videnska 1083, Prague 142 00 Prague 4, Czech Republic

**Keywords:** DMAEA, HEA, hydrolysis, ITC, cytotoxicity, reactivity ratios

## Abstract

Synthetic polycations are key components for engineering polyelectrolyte complexes with wide-ranging biomedical potential. However, the high cytotoxicity of fully charged polycations remains a major limitation for clinical applications. To address this challenge, we report on polycations derived from the cationic monomer 2-(*N,N*-dimethylaminoethyl) acrylate (DMAEA), which gradually loses charge through hydrolysis, thereby reducing their charge density over time. The overall charge fraction (from 100% to 20%) was further controlled through copolymerization with the neutral comonomer 2-hydroxyethyl acrylate (HEA). The selected conditions of reversible addition–fragmentation chain transfer (RAFT) copolymerization, specifically protonation of DMAEA with trifluoroacetic acid to mask its tertiary amino groups, enabled a precise control over the characteristics of the copolymers (termed D/H) up to 75% conversions, with close agreement between theoretical and experimental molecular weights up to 100 000 g/mol, consistently low dispersities (<1.2), and an excellent match between the theoretical and actual copolymer compositions. Hydrolysis studies at pH 7.4 showed that increasing the HEA content in D/H copolymers from 20 to 50 mol % led to only a 10% increase in the hydrolysis over 3 weeks. Isothermal titration calorimetry analysis demonstrated that all copolymers retained their ability to complex with heparin, with binding strength comparable to that of commonly used polycations. Importantly, the cytotoxicity of D/H copolymers toward human umbilical vein endothelial cells (HUVECs) decreased with increasing HEA content, reaching more than 80% cell viability at a relatively high concentration of 30 μg/mL. These findings demonstrate that D/H copolymers combine precise structural control with reduced cytotoxicity, making them promising candidates for biomedical polyelectrolyte platforms.

## Graphical abstract

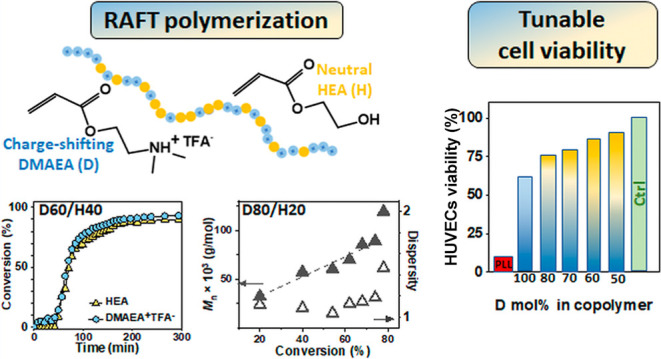

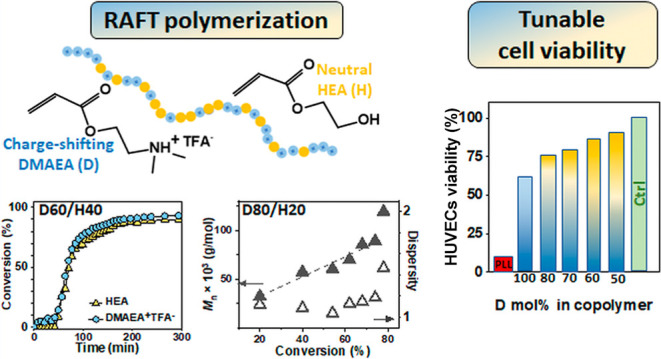

## Introduction

Polycations, polymers containing positive charges in their side chains, can form self-assembled structures with anionic polymers through electrostatic interactions, a phenomenon widely used in gene delivery systems.^1–3^ Similar interactions can also be employed in the formation of ultrathin polyelectrolyte multilayer films, which have potential applications in the construction of biomaterial coatings or biosensors.^4–8^ In such systems, controlled disassembly of self-assembled structures is often desirable. For this purpose, various types of stimuli are employed, such as changes in pH, light, temperature, or ionic strength.^9–12^ An alternative strategy for destabilizing polyelectrolyte complexes involves the use of so-called “charge-shifting” polymers, in which the overall net charge progressively decreases by hydrolysis or reductive degradation under mild conditions, a concept that has attracted growing research interest as a general design strategy.^2,13,14^ Lowering the effective charge density can weaken interpolymer binding and thereby facilitate complex destabilization, which can be advantageous for time-dependent release of associated bioactive cargo.

2-(*N,N*-dimethylaminoethyl) acrylate (DMAEA), the acrylic analogue of the widely used cationic methacrylate, is promising for the formation of “charge-shifting” polycations due to the hydrolysis of ester bonds in side chains bearing tertiary amino-groups. This process leads to a gradual loss of charge along the polycation chain over time and may weaken or even disrupt established electrostatic interactions in the self-assembled structures.^15–20^ Such time-dependent reduction in charge density is particularly relevant for biological applications, as the overall charge content governs polycations cytotoxicity and immunogenicity.^21,22^ Highly charged polycations exhibit strong interactions with negatively charged cell membranes, leading to membrane destabilization and cytotoxicity.^23–26^ Consequently, controlling charge distribution along the polymer backbone, through either hydrolysis, copolymerization with uncharged comonomers, or their combination, represents an effective strategy to balance biological compatibility and functional complexation ability. Copolymers of ionic and neutral units have attracted significant attention as they enable the design of structures with a “diluted” charge content that remains sufficient for self-assembly with counter-polyelectrolytes, but with markedly lower cytotoxicity than their fully charged analogues.^26,27^ Here, we report on the synthesis of well-defined copolymers of DMAEA and 2-hydroxyethyl acrylate (HEA) with variable charge density and investigate their key physicochemical properties relevant to such applications, focusing on the hydrolysis behavior, complexation thermodynamics, and cytocompatibility.

DMAEA-based homo- and copolymers are commonly synthesized by free-radical polymerization;^16,17,28^ however, this strategy cannot guarantee control over the composition and molecular weight (MW) characteristics of the final product. Therefore, controlled polymerization techniques appear to be much more promising. Nevertheless, the synthesis of well-defined polymers based on DMAEA is challenging because of the intrinsic instability of the ester bonds, which are susceptible to both hydrolysis and transesterification. As a result, the reaction conditions must be properly adjusted, for example, by avoiding aqueous conditions and, if necessary, conducting the synthesis under strongly acidic conditions to suppress base-catalyzed hydrolysis.^16^ Similarly, the use of organic mixed solvents free of methanol is required since methanol may act as a nucleophile, thus promoting transesterification of the side chains. In addition, the tertiary amino group of the DMAEA monomer can disrupt the equilibrium of the catalytic system in atom-transfer radical polymerization (ATRP)^29,30^ and can trigger decomposition of the chain-transfer agent (CTA) during reversible addition–fragmentation chain-transfer (RAFT) polymerization, as demonstrated by Li et al.^31^ and more recently by our group.^32^

The RAFT polymerization has been employed to synthesize DMAEA homopolymers of lower MWs, up to approximately 15 000 g/mol, mostly studied for nucleic acid delivery applications.^33,34^ Regarding copolymers, DMAEA has been polymerized with comonomers such as styrene,^31^ methyl acrylate,^35^ HEA,^36^ and several monomers containing protected or nonprotected amino-group such as 2-(*tert*-boc-amino)­ethyl acrylate,^37^ (3-aminopropyl)­methacrylamide,^16,38^ or 2-(dimethylamino)­ethyl 2-(hydroxymethyl) acrylate.^17^ The majority of those studies were carried out in organic solvents, such as dimethylformamide, using trithiocarbonate (TTC)-based CTAs, and the resulting polymers reached MWs around 10 000 g/mol. Alternatively, in the works by Ros et al.,^16,17,38^ an acidified dioxane–water mixture was employed as the solvent, and the monomer was protonated to prevent hydrolysis. Under these conditions, the polymerization based on a dithiobenzoate CTA produced polymers with higher MWs, reaching up to 30,000 g/mol.

The overall properties of DMAEA-based copolymers depend on multiple factors such as molecular weight characteristics, chain architecture, and charge density. Among these, the copolymer composition plays the key role. To determine it, the reactivity ratios of the corresponding monomers are the most essential parameter, and, together with the composition of the polymerization feed, directly dictate the final copolymer composition.^28,39,40^ In addition, they offer valuable insight into copolymer sequence distribution,^40^ enabling fine-tuning of the physicochemical properties of the final material. Despite their importance, only a limited number of studies have reported the reactivity ratios of DMAEA with other monomers.^16,17,37^ Without such data, predictive control over the material properties remains limited. Given these gaps in the literature, we aimed to expand on our previous findings.

Recently, we reported a simple, straightforward protocol for the homopolymerization of DMAEA under nonaqueous conditions using TTC-based CTAs.^32^ Protonation of the tertiary amino groups of the monomer units by trifluoroacetic acid (TFA) prior to polymerization ensured the stability of the CTA during the polymerization, which allowed us to achieve significantly higher MWs than those previously reported in the literature while still maintaining the controlled character of the polymerization. The broad range of well-defined products (10,000–100,000 g/mol) suggests that this polymerization system may be suitable for the copolymerization of DMAEA with various comonomers.

In the present work, we extended our investigation to the synthesis of copolymers of DMAEA, fully protonated with TFA (DMAEA^+^TFA^–^), and the neutral comonomer HEA. HEA was selected to tailor the density of charged side groups along the polymer backbone. From an application perspective, we targeted MWs of approximately 100,000 g/mol, which are favorable, e.g., for forming multilayered polyelectrolyte films due to the increased stability of such complexes. At the same time, DMAEA/HEA copolymers are nondegradable in vivo, and such long chains may remain in the body after disintegration of polyelectrolyte assemblies. Therefore, for the polymerization, we used a bifunctional butane-1,4-diylbis­(4-cyano-4-[(dodecylsulfanylthiocarbonyl)­sulfanyl] pentanoate) (TTC-bis) as CTA, which contains central hydrolytically labile ester bonds. Upon midchain hydrolysis (e.g., under physiological conditions),^41^ the linear polymer chain cleaves into two fragments of approximately half the original MW, which may facilitate removal from the body via renal excretion. The structures of DMAEA^+^TFA^–^ and HEA monomers and TTC-bis are shown in Scheme 1.

**Scheme 1:**
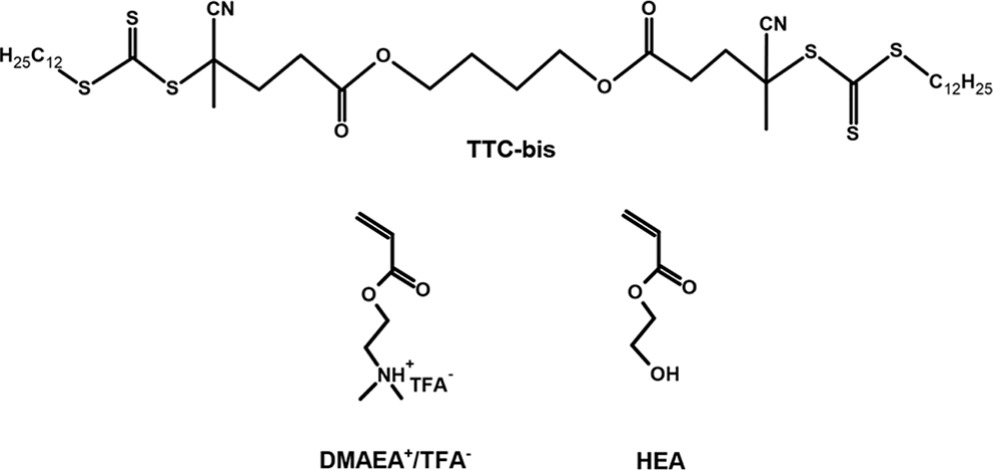
Structure of the Monomers DMAEA^+^/TFA^–^ and HEA and the Chain Transfer Agent, Butane-1,4-Diylbis­(4-Cyano-4-[(Dodecylsulfanylthiocarbonyl)­sulfanyl]­pentanoate) (TTC-Bis), Whose Central Ester Bonds Are Hydrolytically Labile at pH 7.4

By varying the feed composition, we aimed to determine the copolymerization parameters (i.e., the reactivity ratios) of DMAEA^+^TFA^–^/HEA copolymers (abbreviated as D/H) using in situ monitoring of the polymerization via ^1^H NMR-spectroscopy. In parallel, polymerizations on a bench scale were performed to verify the kinetic results. The copolymers were characterized for their composition using ^1^H NMR spectroscopy and MW values and distributions using SEC. Further, we focused on key properties that are relevant for polyelectrolyte-based bioapplications without addressing the performance of fully formulated, self-assembled delivery systems. Specifically, we (i) analyzed the effect of HEA on the base-catalyzed hydrolysis of DMAEA^+^TFA^–^ in D/H copolymers at a pH range of 5 to 7.4 using ^1^H NMR spectroscopy, (ii) assessed the capacity of the copolymers with varying charge content to form polyelectrolyte complexes with a model polyanion (heparin) by isothermal titration calorimetry (ITC), and (iii) evaluated the effect of the charge content on copolymer cytotoxicity toward HUVECs in vitro.

## Experimental Section

### Materials

2-(*N,N*-Dimethylamino)­ethyl acrylate (DMAEA) was purchased from Merck (Prague, Czech Republic) and distilled under vacuum; the aliquots were sealed in glass ampules under an argon atmosphere and stored at –20 °C prior to use. 2-Hydroxyethyl acrylate (HEA) (Merck, Prague, Czech Republic) was extracted with hexane, stored at −20 °C, and passed twice through a short column of basic alumina to remove the MEHQ stabilizer prior to use. 2,2′-Azobis­(4-methoxy-2,4-dimethyl valeronitrile) (V70, Fujifilms, Wako Chemicals, U.S.A.) was used as received. Poly-l-lysine hydrobromide (PLL, P2636, Merck, Prague, Czech Republic) and polyethylene imine (PEI, Merck, Prague, Czech Republic) were used as received. Polymerization solvents *tert*-butanol and dimethylacetamide (DMA) (Merck, Prague, Czech Republic) were dried over molecular sieves. Trifluoroacetic acid (TFA) was purchased from Merck (Prague, Czech Republic) and was used as received. *Tert*-butanol-*d*_10_, acetone-*d*_6_ (99.9% D), chloroform-*d* (99.8% D), D_2_O (99.9% D), and trifluoroacetic acid-*d*_1_ were products of Eurisotop (Sant-Aubin, France).

Butane-1,4-diylbis­(4-cyano-4-[(dodecylsulfanylthiocarbonyl)­sulfanyl]­pentanoate) (a bifunctional CTA) was synthesized according to the procedure described earlier.^41^ The purity of the CTA was verified by ^1^H NMR and HPLC analyses (Figures S1 and S2).

### RAFT Copolymerization of Protonated 2-(*N,N*-dimethylamino)­ethyl Acrylate (DMAEA^+^TFA^–^) and 2-Hydroxyethyl Acrylate (HEA)

General copolymerization procedure: A solution of the DMAEA and HEA monomers in a *tert*-butanol/DMA mixture (70/30 (v/v)) at a total concentration of 2 M was placed in a sealed flask and degassed under a stream of argon for 20 min. TFA (10 mol % excess relative to DMAEA) was then added to the polymerization mixture, and the mixture was stirred for 10 min in an ice bath to protonate the amine groups of the DMAEA side chains. Subsequently, a stock cosolution of CTA and V70 initiator in DMA at a CTA/V70 molar ratio of 5 was added to the flask. The monomer/CTA molar ratio was varied from 130 to 700 to control the MW of the copolymers. The polymerization was conducted at 40 °C for 75–90 min and terminated by rapid cooling in a dry ice bath. The products were purified by dialysis and ultrafiltration in acidified water (pH 3–3.5) using membranes with MWCOs specified in the Supporting Information, and isolated by lyophilization. A representative detailed experimental procedure, including purification and isolation protocols, is described in the Supporting Information.

The determinations of monomer conversions (illustrative ^1^H NMR spectrum of polymerization mixtures shown in Figure S3) and the composition of isolated copolymer products (illustrative ^1^H NMR spectrum shown in Figure S4) were determined in acetone-*d*_6_ by using a Bruker Avance Neo 400 spectrometer operating at 400.1 MHz. The weight-average molecular weight (*M*_w_), number-average molecular weight (*M*_n_), and dispersities (*D̵*) were measured by SEC on an HPLC system (Shimadzu, Japan) with a 0.1 M NaNO_3_/TFA mobile phase (pH 2.5). Further details of the ^1^H NMR and SEC analyses are provided in the Supporting Information.

For clarity, DMAEA^+^TFA^–^/HEA copolymers are hereafter termed D/H copolymers, where the numbers following the monomer abbreviations denote the molar percentage of each monomer in the polymerization feed.

### In Situ Monitoring of RAFT Polymerization by ^1^H NMR Analysis

The polymerization mixtures of DMAEA^+^/TFA^–^ and HEA monomers were prepared using deuterated *tert*-butanol-*d*_10_, DMA, and trifluoroacetic acid-*d*_1_ as polymerization solvents in tear-shaped flasks, following the same protocol as that described for the standard polymerization. Monomer ratios in the range from 0:100 to 100:0 mol % were used for individual experiments, with a fixed monomer/CTA ratio of 450. The polymerization mixture was then transferred to an NMR tube filled with argon. The tube was sealed and placed in the NMR instrument for the analysis of polymerization kinetics at 40 °C.

^1^H NMR in situ polymerization kinetics spectra were acquired with a Bruker Avance III 600 spectrometer operating at 600.2 MHz at 313 K. The width of the 90° pulse was 18 μs, the relaxation delay was 10 s, and the acquisition time was 2.18 s with 2 scans. Kinetics measurements were conducted for 3 h, with time points every 5 or 7 min. Figure S5 presents high-resolution ^1^H NMR spectra of a DMAEA^+^TFA^–^ and HEA polymerization solution recorded at three polymerization time points, including peak assignments of proton signals used to calculate monomer conversions. The conversion of both monomers was calculated from the ratio of the integral intensities of the CH_2_ double bond signals of monomers (*a*_1_ for HEA; *a*_1_’ for DMAEA^+^TFA^–^) and the CH_2_ groups adjacent to the oxygen in the monomer and polymer (d and D for HEA; d′ and D′ for DMAEA^+^TFA^–^). Because the signal positions of the CH_2_ groups in the monomers and polymers (d, D, and d′, D′) are almost identical, the total integral intensity of these signals (i.e., d + D and d′ + D′) was set to 2. Monomer conversions were then calculated using the equations

$$X\left(HEA\right)\left(\%\right)=100\times \left(1-{Ia}_{1}\right)$$

or

$$X\left({DMAEA}^{+}{TFA}^{-}\right)\left(\%\right)=100\times \left(1-{Ia^{\prime }}_{1}\right)$$

The integrated intensities were determined with TopSpin version 4.0.5 software.

### Determination of Reactivity Ratios of DMAEA^+^TFA^–^ and HEA Monomers

The reactivity ratios were determined based on in situ monitoring of the polymerizations by ^1^H NMR spectroscopy, using a Bruker Avance III 600 spectrometer, as described above. A series of copolymerizations was carried out using feed mixtures containing 9, 15, 25, 35, 40, 43, 50, 65, and 85 mol % of HEA to provide a sufficient range of DMAEA^+^TFA^–^/HEA monomer ratios for the reliable determination of reactivity parameters. The mole fractions of monomer 1 (DMAEA^+^TFA^–^), *F*_1_, and monomer 2 (HEA), *F*_2_, incorporated into the forming copolymer were calculated using the following formulas

$$F_{1}=\frac{\left(f_{1}X_{1}\right)}{\left(f_{1}X_{1}+f_{2}X_{2}\right)}$$

and

$$F_{2}=1-F_{1}$$

where *f*_1_ and *f*_2_ = 1 – *f*_1_ are the mole fractions of monomer 1 and monomer 2 in the feed, and *X*_1_ and *X*_2_ are the conversions of monomer 1 and monomer 2, respectively.

The calculated mole fractions *F*_1_ and *F*_2_ were then used to determine the reactivity ratios of DMAEA^+^TFA^–^ and HEA by fitting the data to the Mayo–Lewis terminal model equation

$$F_{1}=\frac{\left(r_{1}{f_{1}}^{2}+f_{1}\left(1-f_{1}\right)\right)}{\left({{r_{1}f}_{1}}^{2}+2f_{1}(1-f_{1})+r_{2}{\left(1-f_{1}\right)}^{2}\right)}$$

where *F*_1_ is the mole fraction of monomer 1 in the copolymer, *f*_1_ is the mole fraction of monomer 1 in the feed, *r*_1_ is the reactivity ratio of monomer 1, and *r*_2_ is the reactivity ratio of monomer 2. In accordance with IUPAC recommendations, nonlinear least-squares fitting was used to directly estimate *r*_1_ and *r*_2_ values, minimizing distortion and bias associated with data transformation.^42^ The experimental data were fitted using Origin’s nonlinear curve fitting tool, set to the Levenberg–Marquardt algorithm. Fitted values *r*_1_ and *r*_2_ were reported along with standard errors and the coefficient of determination *R* ^2^.

To account for a possible drift in feed composition during polymerization, particularly relevant at non-negligible conversions, the instantaneous feed compositions were determined at selected conversions. To obtain a more robust dataset, each sample contributed values determined at several time points, corresponding to overall conversions in the range of 15–40%. This approach allowed for a more accurate calculation of the copolymer composition and improved the reliability of the reactivity ratio estimation.

### Hydrolysis of DMAEA^+^/TFA^–^ Monomeric Units in D/H Copolymers Determined by ^1^H NMR Analysis

3.5 mg of the PDMAEA^+^/TFA^–^ homopolymer or D/H copolymers were dissolved in 0.7 mL of phosphate buffer (65 mM total phosphate, NaH_2_PO_4_/Na_2_HPO_4_) containing 88 mM NaCl in D_2_O, prepared for pH 5, 6.5, and 7.4. The copolymer solutions were transferred to NMR tubes, carefully sealed to prevent evaporation, and kept at room temperature (22 °C) for 3 weeks. At predetermined intervals, ^1^H NMR spectra were recorded using a Bruker Avance Neo 400 spectrometer operating at 400.1 MHz. Acquisition parameters included a 90° pulse width of 16.5 μs, a relaxation delay of 10 s, an acquisition time of 3.41 s, and 64 scans.

The representative ^1^H NMR spectra for the D70/H30 copolymer at pH 7.4 recorded at several time intervals are presented in Figure S6. Hydrolysis was calculated from the ratio of the integrated signal at 3.92 ppm, corresponding to the CH_2_ groups adjacent to the oxygen of dimethylaminoethanol (DMAE) as the degradation product, and the sum of this signal and the broad signal with a maximum at 4.45 ppm, corresponding to the CH_2_ protons adjacent to the oxygen of polymers. The integrated intensities were determined with TopSpin 4.0.5 software with an accuracy of ±2%.

The initial DMAEA^+^TFA^–^ concentration (*C*_0_, M) was calculated from the copolymer composition for a 0.5% solution. The concentration at time *t* (*C*_t_, M) was determined from the ratio of hydrolyzed to total DMAEA^+^TFA^–^ units, as measured by ^1^H NMR. The data were plotted as ln­(*C*_t_) versus *t* and fitted to the equation ln­(*C*_*t*_)+ *k*·*t*-ln­(*C*_0_) = 0, yielding the pseudo-first-order rate constants *k* (s^–1^).

### Isothermal Titration Calorimetry

D/H polymers (D100–7 mg/mL, 0.07 mM; D80/H20–9 mg/mL, 0.09 mM; D70/H30–10 mg/mL, 0.1 mM; D60/H40–13 mg/mL, 0.13 mM; and D50/H50–13 mg/mL, 0.13 mM) were titrated to heparin (1 mg/mL, 0.05 mM) in saline/phosphate buffer at pH 6.5 at 25 °C on a MicroCal ITC200 (Malvern Panalytical Ltd., UK). Each titration was performed by a 0.4 μL injection, followed by 19 2-μL injections. The D/H solutions were additionally titrated into a buffer, and the data were corrected to the corresponding heats of dilution. The isotherms were successfully fitted with a one-site model; from this fit, the binding stoichiometry (*n*), binding constant *K*_a_ (M^–1^), binding enthalpy Δ*H* (kJ/mol), and binding entropy Δ*S* (J/mol·K) were obtained. Free energy of binding was calculated from Δ*G* = −R*T*ln*K*_a_ (kJ/mol). All thermodynamic parameters were normalized to 1 mol of heparin.

### In Vitro Biological Evaluation

#### Cell Culture

The human umbilical vein endothelial cells (HUVECs, Gibco, Fisher Scientific, France) were cultured in Human Large Vessel Endothelial Cell Basal Medium (Medium 200) supplemented with Low Serum Growth Supplement and an antibiotic-antimycotic cocktail (Gibco, Fisher Scientific, France) at 37 °C and 5% CO_2_ until passages 3 (see the Supporting Information for details).

#### Real-Time Cell Analysis (RTCA)

Cell adhesion and proliferation were monitored using the xCELLigence real-time cell analyzer SP (ACEA Biosciences, Inc., U.S.A.) in a humidified incubator at 37 °C and 5% CO_2_. HUVECs were seeded in E-plate 96 PET plates (Agilent, China) (3000 cells/well) and allowed to adhere for 24 h before treatment with the polymer solutions of D100, D80/H20, D70/H30, D60/H40, and D50/H50, and poly­(l-lysine) (PLL, a highly cationic reference control) at final concentrations of 1, 10, 30, 100, and 500 μg/mL. Untreated cells served as a positive control. The cell index, reflecting impedance changes linked to cell adhesion, spreading, and attachment, was continuously monitored every 15 min for 96 h by using the RTCA software. Three independent experiments were performed in triplicate. The experimental details are provided in the Supporting Information.

#### Cell Viability/Cytotoxicity Assay

The cytotoxicity of the D/H polycations toward HUVECs was assessed using the resazurin assay. The cells were seeded in 96-well plates (3000 cells/well) and allowed to adhere for 24 h before treatment with the polymer solutions of D100, D80/H20, D70/H30, D60/H40, and D50/H50, and PLL (a highly cationic reference control) at final concentrations of 1–500 μg/mL. After 72 h of incubation, cell viability was measured using the PrestoBlue Cell Viability Reagent, and the results were normalized to untreated control values set at 100%. Three independent experiments were performed in triplicate. The experimental details are provided in the Supporting Information.

#### Immunofluorescence Staining of the von Willebrand Factor and Cell Nuclei Staining

The cells were seeded into 24-well glass-bottom plates (20,000 cells/well) and allowed to adhere for 24 h before treatment with D60/H40, D80/H20, and PLL (a highly cationic reference control) at final concentrations of 10, 30, and 100 μg/mL for 72 h. After fixation with paraformaldehyde, the cells were permeabilized and blocked, and then incubated with antivon Willebrand factor (vWF) primary antibody, followed by Alexa Fluor 546-conjugated secondary antibody mixed with a Hoechst nuclear stain. Images were acquired using an epifluorescence microscope (Olympus IX 71). Cell densities were quantified with ImageJ FIJI and Cellpose software. The details about the stainings are provided in the Supporting Information.

#### Statistical Analysis

The statistical evaluation was conducted using One-way ANOVA with Student–Newman–Keuls multiple comparison test or with Kruskal–Wallis one-way analysis of variance with Dunn’s multiple comparison test.

## Results and Discussion

### RAFT Copolymerization of DMAEA^+^TFA^–^ and HEA

Because DMAEA is prone to hydrolysis, its polymerization is best carried out in organic solvents. We therefore utilized our previously optimized solvent system, consisting of a *tert*-butanol/DMA cosolution.^32^ The volume ratio was set at 70/30 to ensure a good solubility of the resulting polymers over a wide range of monomer ratios and MWs. This solvent system also readily solubilizes both CTA and initiator V70. To prevent degradation of the CTA by DMAEA,^31,32^ 1.1 equiv of TFA (relative to the DMAEA monomer) was added prior to initiation to fully protonate the DMAEA amino groups. A bifunctional TTC-bis CTA containing hydrolytically unstable central bonds was selected to afford polymers with sufficiently high MWs for practical bioapplications, while enabling facile postsynthetic cleavage to approximately half the length under mild physiological conditions.^41^ This cleavage originates from the bifunctional CTA design; although the cleavage of the D/H copolymers was not quantified in this study, the same midchain cleavage mechanism is expected to apply to the D/H copolymers. The combination of solvent choice, monomer protonation, and CTA design provided control over the polymerization, as reflected in the trends in conversion, MW, and *D̵* described below.

To assess the control of the RAFT process and the effect of monomer feed composition on polymerization behavior, we first studied the polymerization kinetics, then the ability to control MW, and finally the impact of varying the HEA content in the feed. For a preliminary bench-scale kinetic study, the comonomer feed contained 80 mol % DMAEA^+^TFA^–^ and 20 mol % HEA (D80/H20), with an [M]/[CTA]/[I] ratio of 500/1/0.2, targeting a theoretical MW of 115,000 g/mol. The polymerization progress was monitored in parallel by ^1^H NMR spectroscopy in acetone-*d*_6_ (see Figure S3 for an illustrative ^1^H NMR spectrum) and SEC analysis of aliquots periodically withdrawn from the polymerization mixture to correlate the evolution of the monomer conversion with the MW and *D̵* (Figure 1). The kinetic plot (Figure S7) shows an induction period of ∼45 min for both DMAEA^+^TFA^–^ and HEA, followed by a rapid, linear increase in conversion over 60 min. Between 45 and 60 min, the kinetics exhibited a pseudo-first-order character, indicating that the concentration of active radicals remained essentially constant during this stage, confirming controlled chain growth under the studied conditions. Thereafter, the polymerization rate gradually decreased until a plateau was reached. The overall conversion reached 80% within 90 min. Importantly, the molar fractions of both unreacted monomers in the feed remained constant during the polymerization, comparable to the initial values (Figure 1A). This suggests statistical copolymerization with the random incorporation of the monomeric units into the polymer chain, a phenomenon further examined by determining the monomer reactivity ratios.

**Figure 1:**
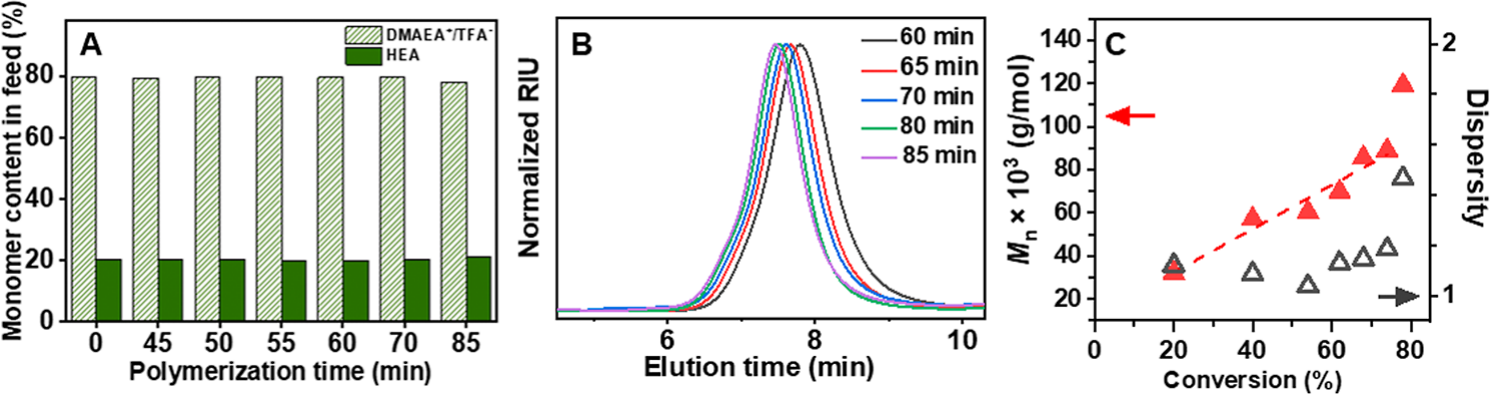
Characteristics of DMAEA^+^TFA^–^/HEA copolymerization of 80% DMAEA^+^TFA^–^ and 20% HEA in the polymerization feed (D80/H20): (A) Content of DMAEA^+^TFA^–^ (dashed green) and HEA (green) in mol % in the polymerization feed at specific polymerization times; (B) SEC chromatography traces of D80/H20 copolymers with increasing polymerization time; (C) dependence of *M*_n_ (red triangle) and *D̵* (empty gray triangle) on the average monomer conversion. Polymerizations conditions: [M]/[CTA] = 500, [CTA]/[V70] = 5, [M] = 2 M in *tert*-butanol/DMA (70/30 (v/v)) solvent mixtures, 40 °C.

SEC chromatograms show the evolution of MWs with an increasing polymerization time (Figure 1B). The chromatograms remained narrow up to ∼70% conversion, after which they gradually broadened toward higher MW. A minor shoulder observed at higher polymerization times beyond 80 min indicates a partial loss of polymerization control at higher conversions, likely due to termination reactions. This observation is consistent with the kinetics data, where the pseudo-first-order character is lost after ∼70% conversion. As shown in Figure 1C, the *M*_n_ increased linearly with conversion up to ∼70%, while *D̵* remained low (*D̵* < 1.2), confirming the well-controlled character of the polymerization up to this point. The corresponding dependence of *M*_n_ and *D̵* on polymerization time is shown in Figure S8.

Based on this observation of well-controlled kinetics, we examined the ability to tailor the MW of the copolymers. At a constant comonomer feed of 80 mol % of DMAEA^+^TFA^–^ and 20 mol % of HEA, the initial [M]/[CTA] ratio was varied from 130 to 700, targeting the MWs ranging from 23,000 to 160,000 g/mol. Keeping the final conversions between 70% and 80%, all products exhibited *D̵* under 1.1, and the determined *M*_n_ values were in good agreement with the theoretical predictions. A detailed summary of these experiments is given in Table 1, and representative SEC traces are shown in Figure S9.

**Table 1:** Effect of [M]/[CTA] Molar Ratio on the Molecular Weight of the Copolymers of DMAEA^+^TFA^–^ and HEA (D/H Copolymers)

	[M]/[CTA]^a^	*M*_n‑theor_ ^b^	conversion^c^	*M*_n‑theor‑conv_ ^b^	*M*_w_ ^d^	*M*_n_ ^d^	*D̵* ^d^
Copolymer	(mol/mol)	(g/mol)	(%)	(g/mol)	(g/mol)	(g/mol)	
D80/H20^e^	130	22,900	86	19,700	21,500	19,900	1.08
	200	45,800	72	32,900	45,300	42,800	1.06
	300	68,700	81	55,600	63,800	61,300	1.04
	500	114,500	72	82,400	97,300	90,100	1.08
	600	120,500	80	96,400	99,500	92,100	1.08
	700	160,300	78	125,000	131,200	123,700	1.06

a M – monomer (the numbers after the monomer abbreviations (D/H) denote the molar % content of monomers in the polymerization mixtures), CTA – CTA agent, [M]/[CTA] – molar ratio of the moles of monomers to moles of CTA.

b *M*_n‑theor_ and *M*_n‑theor‑conv_ theoretical molecular weights based on a [M]/[CTA] molar ratio in the polymerization mixture calculated for a 100% conversion and real conversions that were determined by ^1^H NMR analysis, respectively.

c From ^1^H NMR analysis in acetone-*d*_*6*_. (see Figures S3 and S4 for illustrative ^1^H NMR spectra).

d Weight (*M*_w_) and number (*M*_n_) average molecular weights, and dispersity (*D̵*) of the products, SEC (see the Supporting Information for details about measurement conditions).

e Polymerization time 80 min; [CTA]/[V70] = 5 mol/mol, [M] = 2 M in a *tert*-butanol/DMA (70/30 (v/v)) solvent mixture, 40 °C.

After confirming the controlled character of the polymerization for a fixed monomer ratio and demonstrating the ability to predetermine MW, we investigated the effect of increasing HEA content in the feed on the polymerization kinetics. In these experiments, the reactions were monitored by in situ ^1^H NMR spectroscopy in the *tert*-butanol–*d*_10_/DMA solvent polymerization mixture. The representative ^1^H NMR spectra used for the subsequent analysis are shown in Figure S5. The kinetic data, showing the dependence of monomer conversion on polymerization time for the individual monomers, are presented in Figure 2A,B.

**Figure 2:**
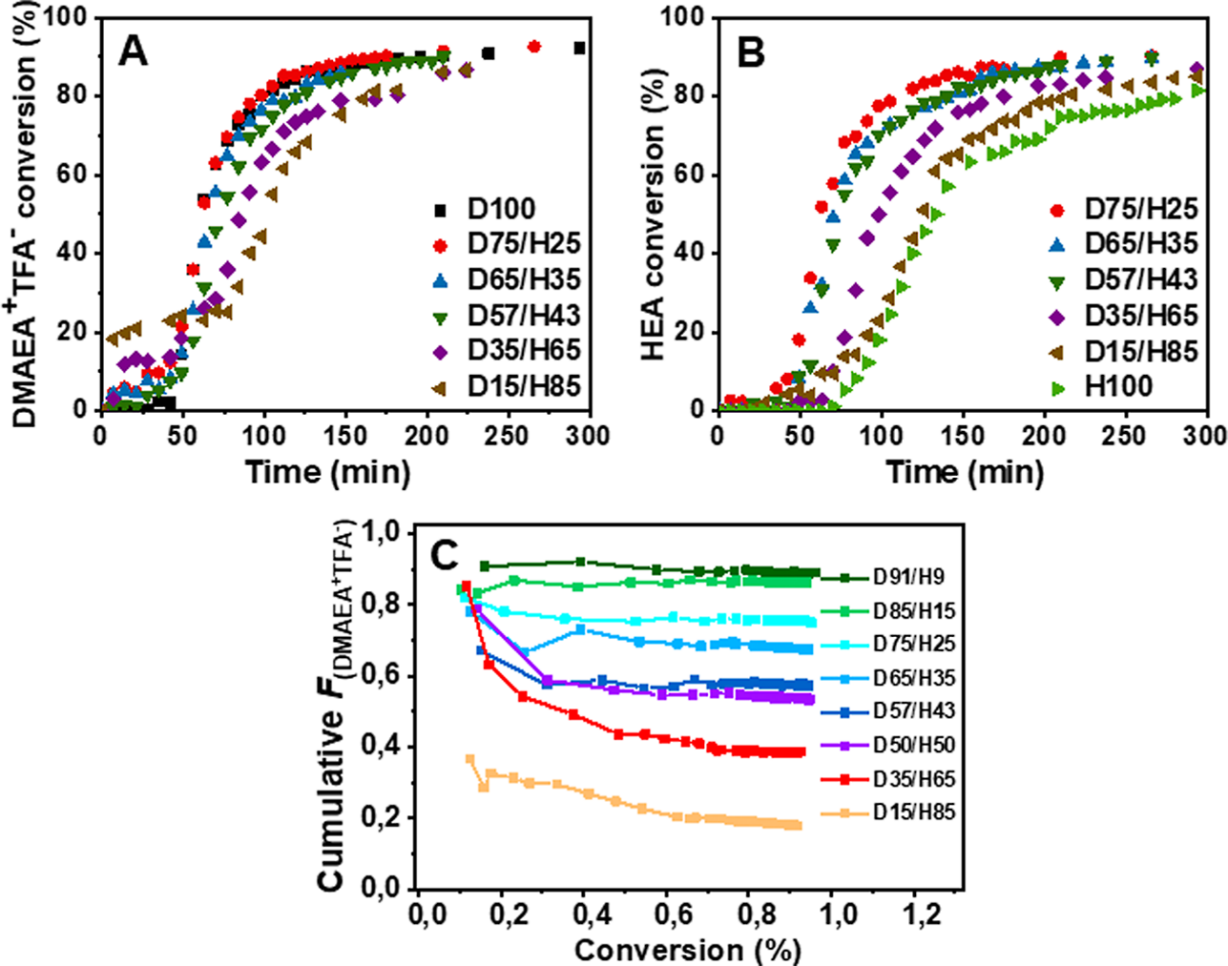
Representative ^1^H NMR kinetic curves of DMAEA^+^TFA^–^/HEA copolymerizations with different monomer contents in the polymerization feed: (A) DMAEA^+^TFA^–^ kinetic curves; (B) HEA kinetic curves; (C) experimental cumulative copolymer composition *F* for DMAEA^+^TFA^–^ units as a function of overall conversion for the feed compositions under study. In the D/H notation, the numbers denote the molar percentage of monomers in the polymerization feed. All polymerizations were performed at [M]/[CTA] = 450, [CTA]/[V70] = 5, [M] = 2 M in *tert*-butanol-*d*_10_/DMA (70/30 (v/v)).

First, the homopolymerization kinetics were compared (Figure S10). ^1^H NMR analysis revealed that the excess of TFA, used to protonate DMAEA prior to polymerization, did not affect the polymerization rate of HEA. Furthermore, the rate of DMAEA^+^TFA^–^ homopolymerization was higher than that of HEA, as evidenced by the steeper slope of the ln­([*M*_0_]/[*M*]) vs time plots, which reflects differences in the chemical structure of the two monomers. The induction period for DMAEA^+^TFA^–^ was approximately half of that observed for HEA. The length of the induction period is closely related to the stability of the formed RAFT adduct radicals, which can be influenced by the polarity of the polymerization medium as well as other medium-dependent factors.^43^ Therefore, an increased fraction of the uncharged HEA monomer in the feed may result in deviations in the copolymerization progress.

The copolymerizations with different monomer feed compositions confirmed this assumption. Figure 2 clearly illustrates that polymerization kinetics strongly depends on the feed composition. As the HEA content in the feed increased, both the overall polymerization rate and the apparent rates of the individual monomers decreased, and the kinetics approached that of HEA homopolymerization. The induction period of DMAEA^+^TFA^–^ remained unchanged across the feeds, while that of HEA gradually decreased with a higher DMAEA^+^TFA^–^ feed content. Notably, HEA is inherently less reactive under the chosen RAFT conditions, but the presence of DMAEA^+^TFA^–^ in the feed accelerates the incorporation of HEA into the copolymer chains. However, the effect diminishes as the HEA fraction increases.

RAFT polymerizations often exhibit an initiation and induction period with nonsteady behavior that can distort calculated reactivity ratios if very low-conversion data are included.^37,43,44^ Therefore, our analysis focused on cumulative compositions between 15 and 40% conversion to achieve reliable parameter estimation. The reactivity ratios obtained from Mayo–Lewis fitting (Figure S11) were *r*_(DMAEA)_ = 1.11 and *r*_(HEA)_ = 2.87, indicating nearly statistical propagation of DMAEA^+^TFA^–^ radicals, while HEA exhibits a moderate tendency toward self-propagation. Statistical RAFT copolymerization, with random copolymer compositions, was reported for the copolymers of DMAEA with 2-(*tert*-Boc-amino)­ethyl acrylate and methyl acrylate in dioxane using TTC-based CTAs.^35,37^ However, the reactivity ratios reported for DMAEA/HEA copolymers synthesized by free radical polymerization in dioxane were 1.2 and 0.9 for DMAEA and HEA, respectively, indicating near-statistical reactivity.^15^ This suggests that the polymerization technique plays a key role in determining the copolymerization behavior of DMAEA^+^TFA^–^ and HEA under the chosen conditions. The similar differences between free radical and RAFT polymerizations have also been shown for the copolymers of *N*-vinylpyrrolidone and isobornyl methacrylate or 2-(dimethylamino)­ethyl methacrylate.^45,46^

Based on the determined reactivity ratio values, it can be assumed that HEA will be consumed faster than DMAEA^+^TFA^–^ at the early stage of polymerization. However, the experimental cumulative composition data for DMAEA^+^TFA^–^ for all feeds (Figure 2C) and data for both monomers for three representative feeds (D85/H15, D50/H50, and D15/H85) (Figure S12) reveal that the actual copolymerization progress does not strictly follow the theoretical prediction. Up to ∼20% overall conversion, DMAEA^+^TFA^–^ is incorporated faster than expected from its feed fraction, while HEA incorporation is delayed, and this delay increases with an increasing HEA content in the feed. The early enrichment likely reflects a kinetic peculiarity of the RAFT system. In HEA-rich feeds, DMAEA^+^TFA^–^ is preferentially consumed during the initial stage of the reaction (up to ∼10–20% conversion, Figure S13), after which its consumption becomes retarded and HEA starts to polymerize. This behavior can be attributed to the RAFT induction period, during which an equilibrium between propagating radicals and dormant chains is being established.^43^ Once the RAFT equilibrium is reached, the system more closely follows the expected reactivity-ratio-driven incorporation, with HEA incorporation increasing at higher conversion levels, especially for HEA-rich feeds.

In addition to the RAFT equilibrium effect, the kinetic penalty associated with HEA propagation must be considered. While the reactivity ratios describe the intrinsic selectivity of chain-end radicals, they do not reflect the absolute rates of propagation under the reaction conditions. HEA homopolymerization is slower in the chosen polymerization medium (Figure S10), most likely due to hydrogen bonding and solvation effects,^47,48^ and this slows the copolymerization as the HEA fraction in the feed increases (Figure 2). As a consequence, at lower HEA contents (≤25 mol %) both monomers polymerize at comparable rates, whereas at higher HEA contents the kinetics become dominated by the slower reactivity of HEA (Figure S13). Similar behavior, although not so pronounced, was reported for RAFT copolymerization of HEA and 2-methoxyethyl acrylate.^49^ The obtained results thus demonstrate that the RAFT copolymerization of DMAEA^+^TFA^–^ and HEA under the chosen conditions cannot be fully described by the terminal model alone since composition-dependent kinetic effects, particularly in HEA-rich feeds, play an important role.

It can be assumed that the microstructure of DMAEA^+^TFA^–^/HEA copolymers is influenced by both the RAFT kinetics and the reactivity ratios. At the beginning of the reaction, there is preferential incorporation of short DMAEA-rich sequences due to the induction period characteristic for RAFT polymerization and the slower propagation of HEA. In the steady-state phase, DMAEA propagates nearly randomly, while HEA radicals form short HEA-rich clusters within the chain. As the conversion increases, the depletion of HEA causes the incorporation of DMAEA^+^TFA^–^ to be favored even more, resulting in the final copolymers with heterogeneous sequence distribution that deviates from the ideal random arrangement.

The effect of HEA content on the copolymer MW and composition was further examined in polymerizations conducted on a bench scale, with the HEA content ranging from 20 to 50 mol %. The polymerization times were selected based on the NMR-derived kinetics and were tuned using bench-scale kinetic verification (i.e., aliquot monitoring) to maintain a control over the polymerization. To minimize termination reactions, the polymerizations were stopped at conversions below 75%. This approach proved to be effective; the measured *M*_n_ values agreed with the theoretical values calculated based on the achieved average conversions, and *D̵* values did not exceed 1.2 (Table 2). Furthermore, the incorporation of both monomers into the polymer chain matched the initial feed, within the experimental uncertainty of the quantitative NMR determination. However, at conversions higher than 75%, SEC analysis showed widening of the chromatographic curves to high MWs with an increase in the HEA content in the feed (Figure S14).

**Table 2:** Effect of the Content of HEA (H) on the Parameters of the Copolymers with DMAEA^+^TFA^–^ (D)^a^

	conversion^b^	copolymer composition^b^ (D/H)	*M*_n‑theor_ ^c^	*M*_n‑theor‑conv_ ^c^	*M*_w_ ^d^	*M*_n_ ^d^	*D̵* ^d^
copolymer code	(%)	(%, mol/mol)	(g/mol)	(g/mol)	(g/mol)	(g/mol)	
D80/H20^e^	75	81/19	114,500	85,900	97,300	90,100	1.08
D70/H30	66	69/31	120,300	79,400	104,800	88,800	1.18
D60/H40	67	58/42	116,400	78,000	80,700	70,900	1.14
D50/H50	65	49/51	106,500	69,200	86,600	73,400	1.18

a The numbers after the monomer abbreviations indicate the molar % content of the monomers in the polymerization mixtures.

b ^1^H NMR analysis of the polymerization mixture and the isolated products in acetone-*d*_*6*_; (see Figures S4 and S5 for illustrative ^1^H NMR spectra).

c *M*_n‑theor_ and *M*_n‑theor‑conv_ are theoretical molecular weights calculated from the [M]/[CTA] molar ratio in the polymerization mixture, based on 100% conversion and real conversions that were determined by ^1^H NMR analysis, respectively.

d Weight (*M*_w_) and number (*M*_n_) average molecular weights, and dispersity (*D̵*) of the products, Size exclusion chromatography (see the Supporting Information for details about measurement conditions).

e Polymerization time 75 min; [M]/[CTA] = 500–600, [CTA]/[V70] = 5 mol/mol, [M] = 2 M in a *tert*-butanol/DMA (70/30 (v/v)) solvent mixture, 40 °C.

In summary, RAFT copolymerizations of DMAEA^+^TFA^–^ with HEA were well controlled up to approximately 75% conversion, as evidenced by the linear *M*_n_–conversion relationship, low dispersities (*D̵* < 1.2), and the absence of high-MW shoulders in SEC traces. At higher HEA contents and/or conversions above 75%, high-MW fractions began to occur, indicating a gradual loss of control under these conditions. Overall, the obtained results demonstrate that the selected solvent system, monomer protonation to prevent the CTA degradation, and CTA design provide robust kinetic control, enabling the precise predetermination of MWs up to 100,000 g/mol. It is worth noting that in previous studies the MW of DMAEA-based copolymers with various comonomers never surpassed 30,000 g/mol.^31,35,37,38^

### Hydrolysis of DMAEA^+^TFA^–^/HEA Copolymers

PDMAEA is known to undergo spontaneous hydrolysis via base-catalyzed ester bond cleavage in aqueous environments, yielding acrylic acid monomer units in the polymer backbone and the small molecule 2-dimethylaminoethanol (DMAE)^19,20^ (for illustration see Figure 3A). Initially, it was reported and widely accepted that the hydrolysis of unprotonated PDMAEA is independent of pH,^20,29,36^ a view still found in recent reports. However, Ros et al.^16^ demonstrated, in studies covering the entire pH range from acidic to alkaline conditions, that the hydrolysis of PDMAEA protonated with HCl is clearly pH-dependent. The previously observed apparent pH-independence of the hydrolysis of unprotonated PDMAEA was most likely caused by the insufficient buffering capacity of the employed buffers with respect to PDMAEA, which can act as a buffer itself. In agreement with these findings, our recent investigations have confirmed that PDMAEA hydrolysis is pH-dependent in both the unprotonated and protonated forms when conditions with adequate buffering capacity are ensured. Moreover, we demonstrated that even at elevated concentrations, acetate buffers (commonly used to maintain pH 5 and 6) are not optimal and should preferably be replaced by phosphate or citrate–phosphate buffers.^32^

**Figure 3:**
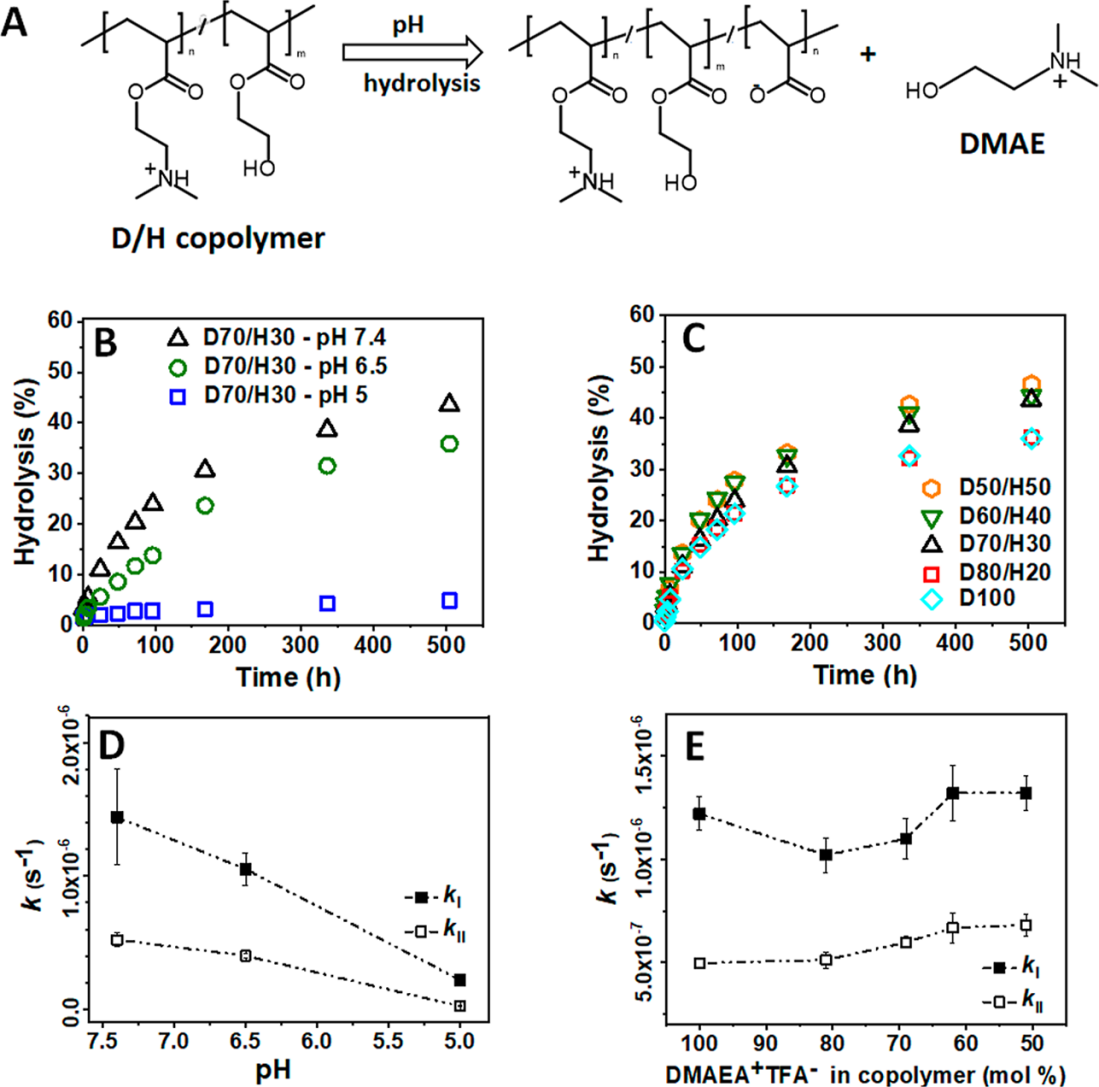
Hydrolysis of 0.5 wt % solutions of DMAEA^+^TFA^–^/HEA (D/H) copolymers with varying contents of charged monomer units at room temperature: (A) Scheme of the pH-dependent hydrolysis of D/H copolymers. (B) Hydrolysis profiles of D70/H30 at pH 5, 6.5, and 7.4. (C) Hydrolysis profiles of D/H copolymers at pH 7.4. Buffers: 0.065 M saline/phosphate-buffered D_2_O at the indicated pH values. The numbers following the monomer abbreviations denote the molar percentage of the monomers in the copolymers. (D) Effect of pH on the initial rate constants *k*_I_ and *k*_II_ for D70/H30 hydrolysis. (E) Effect of the charge content on the initial rate constants *k*_I_ and *k*_II_ at pH 7.4. (Error bars represent a standard deviation of rate constants, derived from the linear fit, see Tables S1 and S2 for numerical values.)

In this work, we investigated the effect of the HEA comonomer on the hydrolysis of DMAEA^+^TFA^–^ in D/H copolymers with up to 50 mol % HEA at pH 5, 6.5, and 7.4. The selected pH range corresponds, to some extent, to the conditions relevant for the processing of PDMAEA^+^TFA^–^-based copolymers into polyelectrolyte complexes intended for bioapplications. First, we confirmed that the hydrolysis of the PDMAEA^+^TFA^–^ homopolymer (D100) increased with increasing pH (Figure S15), reflecting hydroxy-mediated cleavage of ester bonds and thereby verifying previous findings for PDMAEA protonated with hydrochloric acid or TFA.^15,16,32^ Then, the effect of the HEA comonomer on the hydrolysis at pH 5.0, 6.5, and 7.4 was studied by using the D70/H30 copolymer as a representative sample (Figure 3B). Representative ^1^H NMR spectra at pH 7.4 at selected time intervals are shown in Figure S6. The hydrolysis increased with pH in agreement with the behavior observed for the pure D100 homopolymer. This indicates that HEA, as a neutral hydrophilic comonomer, does not alter the pH-dependence of the hydrolysis.

Next, we examined the influence of the HEA content (20–50 mol %) on the degradation of D/H copolymers at pH 7.4, approaching physiological conditions (Figure 3C). Under these conditions, the hydrolysis was comparable for all samples up to ∼48 h of incubation, after which the effect of the HEA content became visible. While a low HEA content in D80/H20 had only a minimal impact, higher HEA contents (i.e., 30–50 mol %) promoted slightly faster degradation compared to the homopolymer, with the differences becoming pronounced after 1 week of incubation. After 3 weeks, the extent of hydrolysis reached approximately 46% for these copolymers compared to ∼36% determined for D100 and D80/H20. Our findings suggest that a higher HEA content slightly enhances the hydrolysis of DMAEA-based copolymers under neutral conditions.

Detailed analysis of the obtained data revealed that the hydrolysis of DMAEA^+^TFA^–^ in D/H copolymers followed a two-step pattern, consistent with the findings of Ros et al.^15–17^ An initial rapid hydrolysis was followed by a pronounced slowdown, reaching a plateau at approximately 45% conversion (Figure 3) after 3 weeks. The data were analyzed using a pseudo-first-order kinetic model, which allowed the determination of the rate constants *k*_I_ and *k*_II_ for the 0–24 h and 24–168 h intervals in the hydrolysis of D70/30 at pH values 5, 6.5, and 7.4 (Figure S16), and for the 0–24 h and 24–98 h intervals in D/H copolymers of varying composition at pH 7.4 (Figure S17).

For the D70/H30 copolymer, the hydrolysis rate decreased markedly with decreasing pH from 7.4 to 5 by nearly 6.5-fold in the first phase and 22-fold in the second phase (Figure 3D, see Table S1 for rate constants). This trend is consistent with the findings of Ros et al., who reported a 10-fold decrease in the rate constant at pH 5 compared to pH 7.^15^ In the case of D/H copolymers, where DMAEA^+^TFA^–^ was diluted with an electroneutral, hydrolytically stable HEA comonomer, our findings show slight deviations from those reported by Ros et al.^15^ The authors reported that HEA fractions below 50% had no influence on the hydrolysis rate and overall hydrolysis course. Pseudo-first-order kinetic analysis in our study also indicates that during the initial stage (0–24 h), the rate constant remained scattered around 1.2 × 10^–6^ s^–1^, without a clear trend (Figure 3E). But notably, the conversion of DMAEA^+^TFA^–^ was ∼3% higher in D50/50 compared to D100 (Table S2). However, during the second stage, the rate constant displayed a tendency to increase with increasing HEA content, accompanied by differences in conversion of up to 10% at the end of the observation period. These results demonstrate a small but distinct influence of the HEA content on DMAEA^+^TFA^–^ hydrolysis, even below 50 mol % of HEA in the D/H copolymer, which can most likely be attributed to indirect macromolecular effects. In particular, incorporation of 30–50 mol % HEA is likely sufficient to reduce the local density of DMAEA cationic groups and thus decrease electrostatic crowding. This reduction may enhance chain solvation and mobility, thereby increasing the accessibility of ester linkages to nucleophilic attack.

Two mechanisms have been proposed to explain the self-inhibited hydrolysis of DMAEA in polymers at neutral to basic pH: (i) steric hindrance arising after macromolecular collapse, caused by interactions between protonated DMAEA groups and carboxyl groups formed upon hydrolysis, and (ii) electrostatic repulsion of OH^–^ ions by negatively charged carboxylates in proximity to the ester bond. The first mechanism was challenged by hydrolysis experiments at high ionic strength (0.5 M NaCl),^16^ which showed no measurable effect on the hydrolysis rate. In contrast, the second mechanism is supported by the observation that DMAEA–acrylic acid copolymers exhibit significantly lower hydrolysis rates compared with DMAEA–HEA copolymers.^15^

### Effect of Charge Content on the Complexation of D/H Copolymers with Heparin in Solution

Polyelectrolyte complexes, which may exist in the form of liquid droplets, nanoparticles, or ultrathin layer-by-layer film coatings, represent a well-established approach for designing carriers that release biologically active molecules in tissue engineering and biomaterials applications. For the controlled and reproducible preparation of such complexes, parameters including MWs as well as the amount and distribution of charges along the polymer backbone are key parameters. RAFT polymerization of protonated DMAEA and its copolymers with HEA, described above, provides the control over these structural features.

In this part of the study, we employed ITC to examine how the gradual replacement of tertiary DMAEA^+^TFA^–^ units by neutral HEA units, (i.e., a reduction in the effective charge density along the polycation chain) affects the polycation ability to form polyelectrolyte complexes with heparin. In this context, ITC provides quantitative information about the binding affinity of the two polyelectrolytes (*K*_A_), the stoichiometric ratio of the bound components (*n*), and how the free energy of binding is partitioned between enthalpic and entropic contributions (Δ*H* and Δ*S*). Heparin was chosen as a model polyanion because of its well-documented bioaffinity for numerous growth factors and cytokines,^50^ which makes it an attractive component for the design of bioactive polyelectrolyte complexes. Further, the hydrolysis studies revealed that cleavage of side-chain amino groups in D/H copolymers was slower in the saline/phosphate buffer at pH 6.5 than that at pH 7.4 (Figure 3A). Therefore, all experiments were conducted in buffer at pH 6.5, a condition that remains compatible with protein stability.

The freshly dissolved copolymers were titrated to heparin within 1 h to ensure that negligible hydrolysis of DMAEA^+^TFA^–^ occurred during the experiment time scale. The titration isotherms in Figure 4 show an increase in enthalpy amplitude and a shift in the D/H copolymer-to-heparin molar ratio to lower values with increasing fractions of the DMAEA^+^TFA^–^ comonomer. This behavior reflects the enhanced capacity for heparin binding at higher charge contents in D/H copolymers. To compare the binding abilities quantitatively, the thermodynamic parameters for D/H–heparin complexation were recalculated per mole of heparin (Table 3). The number of bound heparin molecules increased from ∼4 for D50/H50 up to ∼10 for D100, and the binding constant increased by about an order of magnitude with a higher DMAEA^+^TFA^–^ content.

**Figure 4:**
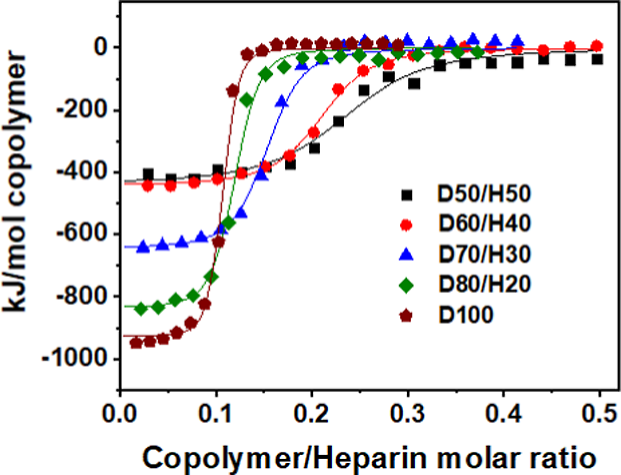
Titration isotherms of DMAEA^+^TFA^–^/HEA copolymer solutions to a heparin solution (ITC, saline/phosphate buffer pH 6.5). The numbers following the monomer abbreviations indicate the molar percentage content of the monomers in the polymerization mixtures.

**Table 3:** Thermodynamic Parameters of the Interaction of Heparin with DMAEA^+^TFA^–^/HEA (D/H) Copolymers of Various Contents of Positively Charged DMAEA^+^TFA^–^ Monomer Units (ITC Evaluation)^a^

sample	*n*	*K*_A_·10^6^ M^–1^	Δ*H* kJ/mol	Δ*S* J/mol·K	Δ*G* kJ/mol
D50/H50	4.3	0.65	–101.7	–229.7	–33.2
D60/H40	5.0	1.52	–88.7	–179.9	–35.3
D70/H30	7.0	1.80	–93.7	–194.1	–35.7
D80/H20	9.0	2.86	–93.3	–189.1	–36.8
D100	10.0	8.35	–92.9	–178.7	–39.5

a The numbers after the monomer abbreviations indicate the molar % content of the monomers in the polymerization mixtures. *n*—binding stoichiometry (a number of heparin molecules bound to the copolymer molecule), *K*_A_—binding affinity constant, Δ*H*—binding enthalpy, Δ*S*—binding entropy, Δ*G*—Gibbs free energy of binding.

The obtained data clearly show that the binding strength and stoichiometry increase with charge density, indicating that the dominant driving force for complexation is electrostatic. The strongly exothermic binding enthalpy, together with the negative entropy change, indicates an enthalpy-driven process. The entropy-driven counterion release, which is intrinsic to polyelectrolyte condensation,^51^ is accompanied by coacervate phase separation as well as salt and water partitioning between the two phases. These processes contribute substantially to the exothermic signal measured by ITC. However, since the interaction stoichiometry clearly corresponds to the charge compensation ratio, the measured enthalpy can be reliably used as a marker enthalpy for determining binding constants.

The determined binding constants were in the 10^6^–10^7^ M^–1^ range, which places D/H copolymer–heparin interactions among strong biomolecular bindings. Compared with other polycation–heparin complexes, the binding strength of D/H copolymer–heparin complexes is comparable with values reported by our group for quaternized chitosan^52^ and dextran,^53^ and for synthetic polycations frequently studied for their polyelectrolyte complexes, such as PLL and polyethylenimine (PEI), also determined in our study (Table S3, and see Figure S18 for titration isotherms). Importantly, even the least charged D50/H50 ligand demonstrated its capability to form stable complexes with heparin.

Overall, the obtained results show that charge density is the decisive parameter controlling complexation strength, while the mechanism remains unchanged across the copolymer series. ITC experiments showed that all D/H copolymers form complexes with heparin, with binding stoichiometry and affinity increasing with the charge content. Even D50/H50 retained the ability to bind heparin, while D100 reached the affinity comparable to those of classical polycations such as PLL and PEI. The interaction was enthalpy-driven, indicating that electrostatics and hydrogen bonding are the dominant forces of complexation.

### Cytotoxicity of D/H Copolymers In Vitro

For bioapplications, materials must exhibit an acceptable level of cytotoxicity. The cytotoxicity of PDMAEA has been studied primarily in the context of its application in DNA delivery, where it was shown to depend on polymer concentration and MWs.^20,33,38,54^ Recent studies further suggest that, due to the progressive hydrolysis of amino groups in the side chains, yielding acrylic acid units and low-molecular-weight DMEA as side products (both classified as nontoxic),^55,56^ the overall toxicity of the polycation decreases over time.

The experiments aimed to evaluate the effect of decreasing the fraction of the positive charge in D/H copolymers on the endothelial cell response. HUVECs were selected as model cells, as they are generally considered more sensitive to chemical and environmental stimuli than fibroblasts, typically employed for standard cytotoxicity screening. In addition, HUVECs are often evaluated by the xCELLigence RTCA system without being negatively affected by the electric field during the measurement. The cells were exposed to increasing concentrations (1–500 μg/mL) of D/H copolymers containing 100% (D100), 80% (D80/H20), 70% (D70/H30), 60% (D60/H40), and 50% (D50/H50) molar fractions of DMAEA^+^TFA^–^ monomer units. PLL was included as a highly cationic reference control. Several complementary methods were employed to assess the effects on proliferation, viability, and morphology during a 3 day culture of HUVECs with the copolymers.

The immediate effects on *cell adhesion, spreading, and proliferation* were monitored using the xCELLigence RTCA system. At 10 μg/mL, the cells treated with all polycations exhibited the normalized cell index values as in the Control (Figure 5A). At 30 μg/mL, the highly charged D100 and D80/H20 reduced the cell index to 60–70%, while D70/H30 and D60/H40 retained cell index values at 71% and 78% of the Control, respectively, indicating moderate to mild toxicity. At higher concentrations, all copolymers except D50/H50 reduced the cell index below 30%. In contrast, D50/H50 showed no detectable cytotoxicity, likely consistent with its low charge. The treatment with PLL resulted in low cell index values at 30–500 μg/mL, consistent with its well-documented severe membrane toxicity.^23,25,57^ Tables S4–S7 present detailed statistical analyses of all copolymers across concentrations, with the charge content and concentration as the key parameters.

**Figure 5:**
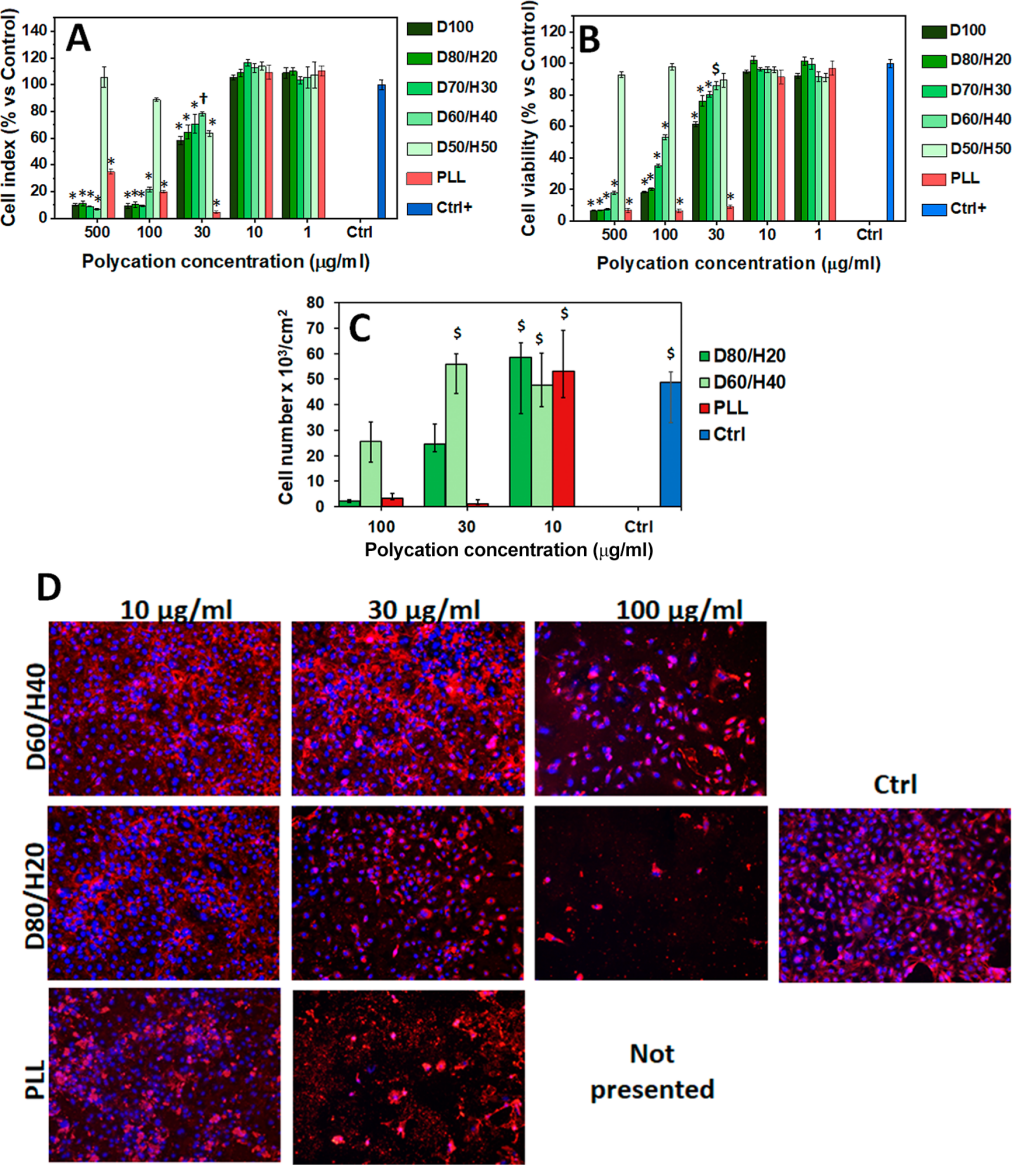
In vitro cytotoxicity of polycationic D/H copolymers with varying charge content toward HUVECs after 3 days of culture. (A) Cell index values of HUVECs. (B) Cell viability assessed using the PrestoBlue assay. Data are expressed as a percentage relative to the positive control (100%) and presented as mean ± SEM (One-way ANOVA with Student–Newman–Keuls multiple comparison test; * indicates *p* < 0.001, † indicates *p* < 0.01, and $ indicates *p* < 0.05 versus the Control; detailed statistical analyses are provided in Tables S4–S7). (C) Cell number of HUVECs calculated from micrographs with nuclei stained by Hoechst dye. Data are expressed as medians, with variability indicated by the 25th and 75th percentiles as lower and upper deviations, respectively. (Kruskal–Wallis one-way analysis of variance with Dunn’s multiple comparison test; $ indicates *p* < 0.01 versus the PLL sample at 100 and 30 μg/mL and versus the D80/H20 sample at 100 μg/mL.) (D) Microscopy images of HUVECs with immunofluorescence staining of von Willebrand factor (red color) and cell nuclei counterstained with Hoechst (blue color) (Olympus IX71 epifluorescence microscope, objective ×10; scale bar = 200 μm). Polycations tested: D100, D80/H20, D70/H30, D60/H40, D50/H50, and PLL (fully charged control). Untreated cells served as the positive control.

The *metabolic activity* assay confirmed these findings (Figure 5B). HUVECs treated with all copolymers at 1 and 10 μg/mL concentrations metabolized comparably to the Control. Then, the cell viability began to decrease at 30 μg/mL, and the polycation toxicity increased proportionally to the charge content from D60/H40 to D100. However, only D100 showed a value below 70% of that of the Control, indicating a moderate reduction in metabolic activity. At 100 and 500 μg/mL, all samples, except D50/H50, markedly reduced the HUVEC viability below 50%. D50/H50 maintained metabolic activity at control levels across all concentrations. In contrast, the PLL treatment led to viability values lower than 15% of the positive control already at 30 μg/mL concentration, demonstrating higher toxicity than that of the fully charged D100. PLL is known to cause dose-dependent cytotoxic, apoptotic, and genotoxic effects, likely through membrane disruption and mitochondrial apoptosis mechanisms.^57,58^

The HUVECs were then cultured with D60/H40, D80/H20, and PLL at concentrations of 10, 30, and 100 μg/mL under *standard culture conditions*. The cell morphology 24 h after copolymer addition is shown in Figure S19. At 10–30 μg/mL, the copolymer-treated cells were spread with a cobblestone-like morphology comparable to the Control, whereas PLL at 30 μg/mL already reduced the cell density. At 100 μg/mL, the cells grew at a low density or, in the case of PLL, were difficult to visualize likely due to adsorption of PLL on the well bottom.

On day 3, the cells were stained for von Willebrand factor (vWF) and nuclei with Hoechst, and cell densities were quantified from micrographs (Figure 5C). The most homogeneous cell distribution and vWF staining were observed for D80/H20 and D60/H40 at 10 μg/mL, for D60/H40 at 30 μg/mL, and in Control (Figure 5D). Because PLL at 100 μg/mL adsorbed on the bottom surface and interfered with the immunofluorescence staining, these samples are not presented. At 10 μg/mL, the cell densities remained comparable to the Control. At 30 μg/mL, the density decreased for the highly charged D80/H20, and PLL almost entirely suppressed the proliferation. At 100 μg/mL, D60/H40 further reduced the cell numbers, and D80/H20 and PLL nearly eliminated cell growth. These findings are consistent with the xCELLigence RTCA data and metabolic activity assay.

The results demonstrate a strong correlation between the polycation charge density and cytotoxicity toward endothelial cells. All copolymers (except D50/H50) almost completely suppressed cell proliferation at 500 μg/mL, consistent with the cell treatment with fully charged polycations such as PEI and PLL.^59^ Compositional effects became apparent already at 100 μg/mL, particularly in the metabolic activity data, where the HUVEC viability increased with decreasing charge content. The polycations containing more than 60 mol % DMAEA^+^TFA^–^ remained strongly cytotoxic, but in contrast to the severe toxicity reported for PEI, PLL, polyallylamine (PAH), or diethylaminoethyl-dextran at comparable concentrations.^23,25^ At 30 μg/mL, the trend became more evident; the copolymers with less than 70 mol % charged units maintained higher cell proliferation and metabolic activity, shifting from moderate toxicity for highly charged polycations to no detectable toxicity for D60/H40 and D50/H50. In contrast, viability values below 40% have typically been reported for PLL and PAH,^58,60^ and were also observed for PLL in our study. The magnitude of this composition effect is quantified in Figure 5A,B across concentrations (and discussed above), and the corresponding statistical evaluation is provided in Tables S4–S7. Furthermore, the treatment with the homopolymer D100 maintained 60% viability after 72 h, whereas the methacrylated analogue poly­(2-(dimethylamino)­ethyl methacrylate) (PDMAEMA), at comparable MW and 30 μg/mL, reduced endothelial cell viability to about 20% after 12 h.^61^ The difference can be attributed to the higher hydrophobicity of PDMAEMA, resulting from the presence of methacrylate groups in the backbone. It has been reported that increased PDMAEMA hydrophobicity, introduced by hydrophobic comonomers, significantly enhanced cytotoxic effects toward different cell types.^26^

The biological response to polymer biomaterials is contingent on the intended application and exposure scenario. When considering the molecular characteristics of polymers, the most relevant parameters include the molar-mass characteristics, the chemical functionality and the associated charge density, the hydrophilicity/hydrophobicity balance, and the chemical or sequence homogeneity of the chains.^26,27,57,62^ In this work, the polymer backbone chemistry as well as the molar-mass characteristics (*M*_w,_ *M*_n_, and *D̵*) were kept comparable across the D/H copolymer samples. The only intrinsic parameter intentionally varied was the DMAEA^+^TFA^–^ content, i.e., the number of ionizable cationic units along the chain. We therefore consider the following parameters as the primary factors contributing to the observed biological response. The first factor is the chemical nature of the *cationic functionality*. It is well established that primary and secondary amines exhibit higher cytotoxicity than tertiary or quaternary amines.^23,26^ This knowledge explains the significantly lower cytotoxicity observed for the D100 homopolymer compared with PLL at 30 μg/mL concentration, as well as with literature data on PLL, PAH, and PEI.^23,25,60^

Second, the overall *charge density* plays a key role. Over the past decades, intensive research on both PLL and PEI has led to numerous strategies to decrease their cationic charge content and, thereby, mitigate the inherent cytotoxicity. These include PEGylation (most often by grafting PEG chains), partial substitution of amine groups with neutral or anionic moieties (e.g., acetylation, succinylation), and copolymerization or conjugation with neutral or biodegradable segments.^57,62^ Consistent with this principle, the D/H copolymers in our study exhibited reduced charge density along the backbone through incorporation of a neutral HEA comonomer, resulting in lower cytotoxicity. A comparable effect was observed for PDMAEMA copolymers with the neutral hydrophilic comonomer triethylene glycol methyl ether methacrylate (TEGMA; 10–50 mol %).^26^ The PDMAEMA/TEGMA copolymers (60 kDa) containing 30 mol % or more TEGMA were classified as nontoxic at 25 μg/mL concentration toward various cell types. A similar trend was also reported for quaternized dextrans, where cytotoxicity toward fibroblasts increased with the degree of substitution (35%–50%).^63^ However, in that study, the cells exposed to quaternized dextran with 50% substitution at a 100 μg/mL concentration for 24 h exhibited only 40% viability, in contrast to the excellent cytocompatibility of the D50/H50 copolymer in our study.

Finally, a relevant structure-dependent parameter is *the hydrolytic stability of the ester linkage in the DMAEA side chain*. The D/H copolymers underwent progressive side-chain hydrolysis, leading to a gradual decrease in amino groups along the polymer chain by ∼20–25% within 3 days in PBS (Figure 3). This ongoing reduction in charge density during the culture is expected to mitigate cytotoxic effects, consistent with the report on improved biocompatibility of nicotinate-based copolymers when the hydrolytically stable *N*-[2-(dimethylamino)­ethyl]­acrylamide comonomer was substituted with DMAEA.^64^ This interpretation is further supported by the findings of Ros et al.^38^ The copolymers of DMAEA and 3-aminopropylmethacrylamide formed polyplexes with 60bp DNA that were better tolerated by HeLa cells as DMAEA content in the copolymers increased. The enhanced cell viability was attributed to the hydrolytic loss of cytotoxic cationic charge from DMAEA units, thereby decreasing the polycation toxicity over time.

Overall, the presented results provide a consistent structure–property–response picture. The selected polymerization protocol enables the reproducible preparation of D/H copolymers with a well-controlled composition and well-defined molar-mass characteristics, including narrow molar-mass distributions across the D/H copolymer series. The samples were also thoroughly purified to remove low-molecular-weight residues. Based on the reactivity ratio determination (i.e., a near-ideal propagation for DMAEA^+^TFA^–^ and a moderate preference of HEA for self-propagation), the copolymer microstructure is expected to be predominantly statistical within the conversion range used, without pronounced blockiness, thus providing a relatively uniform distribution of charged groups along the chains. Importantly, this level of control minimizes the contributions from low-molecular-weight residues and the sample heterogeneity, which are known risk factors for increased cytotoxicity, and thus represents an important prerequisite for obtaining the polycations with reduced cytotoxicity. Next, the time course of side-chain hydrolysis of DMAEA^+^TFA^–^ units was similar across the D/H copolymer series, indicating that the time-dependent charge loss is comparable across the compositions. This simplifies the comparison of cytotoxicity trends, as hydrolysis contributes to a comparable extent in all copolymer compositions. Consequently, the proliferation and metabolic activity assays show a clear composition- and dose-dependent response, with a progressively improved cell response as the DMAEA^+^TFA^–^ content decreases from 100 to 50 mol %. For example, at 30 μg/mL, HUVEC viability increases with decreasing charge content, from 62% (100 mol %) to 76% (80 mol %), 80% (70 mol %), 86% (60 mol %), and 90% (50 mol %). In contrast, a PLL negative control yielded only 9% viability under the same conditions. Thus, the low toxicity of D70/H30 and D60/H40 copolymers and the cytocompatibility of D50/H50 indicate that decreasing the cationic component modulates the polymer–cell interactions, thereby improving biocompatibility. The ITC results further demonstrate a clear charge-dependent increase in heparin binding strength and stoichiometry across the D/H copolymer series, which complements the cytotoxicity trends and illustrates a tunable balance between the complexation ability and cytocompatibility within the same polymer platform.

## Conclusions

The proposed conditions for RAFT copolymerization of the protonated DMAEA^+^TFA^–^ and HEA monomers allowed a well-controlled synthesis of the D/H copolymers with a wide range of MWs between 20 000 and 100 000 g/mol and low dispersities (<1.2). The process remained well controlled up to ∼75% conversion, with no effect of HEA content on the product characteristics. The obtained kinetic data and reactivity ratios indicate that the charged units are distributed relatively uniformly along the copolymer chain, with local microdomains of DMAEA^+^TFA^–^ or HEA sequences arising also from the RAFT induction period and the kinetic penalty of HEA propagation.

The HEA comonomer did not alter the pH dependence of hydrolysis of D/H copolymers, and the HEA content had only a minor effect on the fast-initial phase. However, over time, the copolymers with more than 20 mol % HEA hydrolyzed faster, reaching ∼45% of hydrolyzed units compared to ∼36% observed for lower-HEA-content products after 3 weeks. Furthermore, ITC experiments revealed that although the complexation capacity of copolymers with heparin decreased as the HEA fraction increased from 20 to 50 mol %, the binding strength remained comparable to that of PLL or PEI, indicating high-affinity interactions. Finally, we demonstrated a direct relation between the cytotoxicity toward HUVECs and the charge density of the D/H polycations. The strong toxicity observed for fully charged D100 and D80/H20 shifted to relatively low toxicity of D70/H30 and D60/H40, and further to the cytocompatibility of D50/H50, proving that decreasing the cationic component enhances polycation biocompatibility.

The proposed polymerization conditions open a pathway to synthetic polycations that retain strong complexation capacity yet exhibit markedly improved cytocompatibility, paving the way for their translation into biomedical applications.

## Supplementary Material



## Notes

Experimental details on RAFT copolymerization, NMR and SEC analyses, and cytotoxicity evaluation; additional results of CTA characterization, ^1^H NMR and SEC analyses of copolymers, kinetic plots for RAFT copolymerizations, reactivity ratios evaluation, extended data on the hydrolysis (^1^H NMR spectra, determination of initial rate constants) and ITC experiments (titration isotherms of PLL and PEI), and depiction of HUVEC morphology after 24 h, including statistical analyses of cell response to D/H copolymers (PDF)
