# Supplementary material for: Charge-Shifting Copolymers of 2‑(N,N‑Dimethylamino)Ethyl Acrylate and 2‑Hydroxyethyl Acrylate via RAFT Polymerization: Balancing the Charge Content and Biological Response
Source: ACS Appl Polym Mater. 2026 Mar 1;8(5):3368–82. doi: 10.1021/acsapm.5c04108 (PMC12993804; doi:10.1021/acsapm.5c04108)
Supplement: Supplementary file 1 [file ap5c04108_si_001.pdf]

THE SUPPORTING INFORMATION

Charge-shifting copolymers of 2-(N,N-dimethylamino)ethyl acrylate and 2-hydroxyethyl acrylate via RAFT polymerization: Balancing charge content and biological response

Radoslava Sivkova[a], *Monika Matiyani*[a], *Gabriela S. García-Briones*[a], *Rafal Konefal*[a,b], *Volodymyr Lobaz*[a], *Lenka Kotrchová*[a,], *Elena Filová*[c], *Natália Podhorská*[a], *Libor Kostka*[a], *Dana Kubies*[a,]*

[a]Institute of Macromolecular Chemistry, Czech Academy of Sciences, Heyrovsky nam. 2, 162 00 Prague 6, Czech Republic

[b]NanoBioMedical Centre, Adam Mickiewicz University, Wszechnicy Piastowskiej 3, 61-614, Poznań, Poland

[c]Laboratory of Biomaterials and Tissue Engineering, Institute of Physiology of the Czech Academy of Sciences, Videnska 1083, 142 00 Prague 4, Czech Republic

*e-mail: kubies@imc.cas.cz

CONTENT

EXPERIMENTAL PART 4

RAFT copolymerization of DMAEA⁺TFA⁻ and HEA 4

^{1}H NMR spectroscopy 5

Size exclusion chromatography analysis 5

Cell culture 6

Live cell behavior monitoring 6

Cell viability/Cytotoxicity assay 6

Immunofluorescence staining of the nucleus and von Willebrand factor 7

RESULTS 8

Figure S1. ^{1}H NMR spectrum of the bifunctional CTA in $CDCl_3$ 8

Figure S2. HPLC curve of the bifunctional CTA. 8

Figure S3. ^{1}H NMR spectrum of the P(DMAEA^{+}TFA^{-}-*co*-HEA) polymerization solution in acetone-d_6 at 75% conversion 9

Figure S4. ^{1}H NMR spectrum of D70/H30 copolymer in acetone-d_6 10

Figure S5. ^{1}H NMR spectra of the DMAEA^{+}TFA^{-}/HEA polymerization solution in *tert*-butanol-d_{10}/DMA (70/30 (v/v)) 11

Figure S6. Representative ^{1}H NMR spectra of the D70/H30 copolymer incubated in saline/phosphate-buffered D_2O (0.065 M) at pH 7.4. 12

Figure S7. Monomer conversion versus polymerization time for the D85/H15 copolymer prepared by bench-scale RAFT polymerization. 13

Figure S8. Dependence of M_n (red triangle) and $Đ$ (empty gray triangle) on the polymerization time for 13

Figure S9. SEC traces of D80/H20 copolymers with increasing [M]/[CTA] ratio. 14

Figure S10. Pseudo-first-order kinetic plots of HEA, HEA/TFA (0.1 mol% relative to HEA), and DMAEA^{+}TFA^{-} homopolymerizations 14

Figure S11. Experimental copolymer composition in DMAEA^{+}TFA^{-} units, $F_{(DMAEA+TFA-)}$, as a function of the DMAEA^{+}TFA^{-} feed fraction, $f_{(DMAEA+TFA-)}$, fitted to the Mayo–Lewis model. 15

Figure S12. Experimental cumulative copolymer compositions, F, for DMAEA^{+}TFA^{-}/HEA (D/H) copolymers for three representative feeds 15

Figure S14. SEC traces of DMAEA^{+}TFA^{-}/HEA copolymers with various comonomer ratios 17

Figure S15. Effect of pH on the hydrolysis of 0.5% wt. solutions of PDMAEA^{+}TFA^{-} homopolymer. 17

Figure S16. Effect of pH on the hydrolysis of D70/H30. 18

Table S1. Initial rate constants k_I and k_{II} of the hydrolysis of D70/H30 copolymer at different pH values 18

Figure S17. Effect of the HEA content on the hydrolysis of D/H copolymers at pH 7.4....................... 19

Table S2. Initial rate constants k_I and k_{II} of the hydrolysis of D/H copolymers 20

Figure S18. Titration isotherms of PLL and PEI solutions 21

Table S3. Thermodynamic parameters of the interaction of heparin with PLL and PEI........................ 21

Figure S19. The morphology and spreading of HUVECs cultured for 24 hours in the presence of polycations. 22

Table S4. Statistical analysis: Effect of the charge content in D/H copolymers on HUVEC cell index at fixed polycation concentrations... 23

Table S5. Statistical analysis. Effect of the polycation concentration on HUVEC cell index at a fixed charge content of D/H copolymers. 24

Table S6. Statistical analysis: Effect of the charge content in D/H copolymers on cell viability at fixed polycation concentrations... 26

Table S7. Statistical analysis. Effect of the polycation concentration on cell viability at a fixed charge content of D/H copolymers. 27

EXPERIMENTAL PART

RAFT copolymerization of $DMAEA^{+}TFA^{-}$ and HEA

Copolymerization procedure ([M1+M2]/[CTA] = 500, [$DMAEA^{+}TFA^{–}$]/[HEA] = 0.65/0.35): A solution of the monomers (325 mg, 2.25 mmol DMAEA, and 140 mg, 1.21 mmol HEA, total 3.46 mmol) in a mixture of 1014 μl *tert*-butanol and 416 μl DMA was placed into a sealed flask and degassed under a stream of argon for 15 minutes. Then, TFA (190 μl, 2.47 mmol; 10% molar excess relative to DMAEA) was added dropwise to the polymerization mixture under an argon atmosphere. Finally, 100 μl of a freshly prepared stock CTA/V70 co-solution (CTA: 5.74 mg, 0.0013 mmol; V70: 0.41 mg, 0.0067 mmol) in DMA was added to the flask using a Hamilton syringe under an argon atmosphere. The flask was immersed in an oil bath, and the polymerization was carried out at 40 °C for up to 3 hours. The polymerization was terminated by rapid cooling in a dry ice bath.

Purification of small-scale preparations: The crude products were purified by five ultrafiltration cycles of the polymerization mixture in water acidified with TFA (pH 3–3.5) using Amicon® Ultra-15 centrifugal filter units (3 kDa, MilliporeSigma). In each cycle, 200 μl of polymerization mixture was added to the filtration cell, diluted with 13 ml of acidified water, and spun at 6000 rcf for 25 min at 10 °C.

Purification of bench-scale preparations: The polymerization mixture was dialyzed for one day against acidified water (pH 3–3.5) using dialysis tubes (3 kDa cutoff) to remove organic solvents. The product solution was then purified by four successive ultrafiltration cycles in an Amicon® Stirred Cell 200 mL equipped with Ultracel® PL 10K membranes, using acidified water (pH 3-3.5) as the medium. If needed, after each cycle, the pH of the newly diluted solution was readjusted to pH 3 before proceeding to the next cycle.

The purified products were isolated by lyophilizing the solutions from plastic containers, since the polymers adhered strongly to glass surfaces, yielding yellowish, rubber-like solids. The efficiency of the purification was verified by ^{1}H-NMR analysis through monitoring the absence of residual monomers. The products were stored in plastic vials at –20 °C.

^{1}H NMR spectroscopy

The determinations of monomer conversions for large-scale polymerizations (100 μL of a polymerization mixture in 0.6 mL of acetone-d_6) and the compositions of copolymer products were conducted in acetone-d_6 using a Bruker Avance Neo 400 spectrometer operating at 400.1 MHz. The width of the 90° pulse was 16.5 μs, the relaxation delay was 10 s, and the acquisition time was 3.41 s, with 32 scans.

An illustrative ^{1}H NMR spectrum with peak assignments of proton signals used to calculate monomer conversions is shown in **Figure S3.** The conversion of both monomers was calculated from the ratio of the integral intensities of the CH_2 double bond signals of monomers (a_1 for $DMAEA^+TFA^-$; a_1’ for HEA) and the CH_2 groups adjacent to the oxygen in monomer and polymer (d and D for $DMAEA^+TFA^-$; d’ and D’ for HEA). Because the signal positions of the CH_2 groups in the monomers and polymers (d, D, and d’, D’) are almost identical, the total integral intensity of these signals (i.e., d + D and d’ + D’) was set to 2. Monomer conversions were then calculated using the equations:

$$\text{Conv. DMAEA}^+\text{TFA}^- (\%) = 100 \times (1\text{-I}a_1) \quad \text{or} \quad \text{Conv. HEA} (\%) = 100 \times (1\text{-I}a_1')$$

The copolymer composition (from isolated products) was calculated from the ratio of the integrated signals corresponding to the CH_2 groups adjacent to the oxygen at 4.46 ($DMAEA^+TFA^-$) and 4.21 (HEA) ppm to the integrated signal at 2.50 ppm attributed to the CH groups of the copolymer backbone (**Figure S4**). The integrated intensities were determined with TopSpin 4.0.5 software with an accuracy of ± 2%.

Size exclusion chromatography analysis

Weight-average molecular weight (M_w), number-average molecular weight (M_n), and dispersities (*Đ*) were determined by SEC on an HPLC system (Shimadzu, Japan) equipped with an internal UV-VIS diode array detector (SPD-M20A), external differential refractometer (Optilab T-rEX), and multiangle light scattering detector (DAWN HELEOS II, both Wyatt Technology, USA). Gram precolumn + TSKgel G3000PWXL-CP (7.8 x 300 mm, Chromservis s.r.o., Prague, Czech Republic) and a mobile phase of 0.1 M $NaNO_3$/TFA, pH 2.5, at a flow rate of 1 mL/min were used to analyze the charged samples. A method based on a known total injected mass assuming 100%

recovery was used to estimate the d*n*/d*c* value (0.102 mL/g) to calculate the MWs from the light scattering data.

Cell culture

The human umbilical vein endothelial cells (HUVEC) were obtained from Gibco (Gibco, Fisher Scientific, France). Cells were cultured in Human Large Vessel Endothelial Cell Basal Medium (Medium 200) supplemented with Low Serum Growth Supplement and an antibiotic-antimycotic cocktail (1% Streptomycin and 0.1% Penicillin) (Gibco, Fisher Scientific, France) at 37 °C and 5% CO_2. The cells in passages 3 were used for all experiments.

Live cell behavior monitoring

Real-Time Cell Analysis (RTCA) of cell adhesion and proliferation was performed using the xCELLigence real-time cell analyzer SP (ACEA Biosciences, Inc., U.S.A.) in a humidified incubator at 37 °C and 5% CO_2. First, 160 µl of cell culture medium was added to each well of a 96-well E-plate PET plate (Agilent, China), and the chambers were calibrated. Next, 20 µl of HUVEC cells in culture medium were added to each well, at a seeding density of 3 000 cells per well, and the cells were allowed to adhere for 24 hours. After this incubation, 20 µl of stock solutions of D/H copolymers containing 100% (D100), 80% (D80/H20), 70% (D70/H30), 60% (D60/H40), and 50% (D50/H50) molar fractions of protonated, charged DMAEA^{+}TFA^{-}(D) units, and poly(L-lysine) (PLL, P2636, Sigma Aldrich, Prague, Czech Republic, a negative control) in the medium was added to the wells to obtain final copolymer concentrations of 1, 10, 30, 100 and 500 µg/mL. Untreated cells served as a positive control. The cell index, a parameter of impedance that depends on the number of adhered cells, cell spreading, shape, and the quality of cell-substrate attachment, was then continuously monitored at 15-minute intervals for 96 hours, with data acquisition performed using the RTCA software. Three independent experiments with three parallels were performed, and data were expressed as a percentage relative to the positive control (100%).

Cell viability/Cytotoxicity assay

Cytotoxicity of the D/H polycations of various charge content to HUVECs was assessed using the resazurin assay as follows: First, 160 µl of cell culture medium and 20 µl of HUVEC cells in culture medium (3,000 cells/well) were added into each well of a 96-well plate, and the cells were allowed

to adhere for 24 hours. Next, 20 µl of the stock solutions of D/H copolymers containing 100% (D100), 80% (D80/H20), 70% (D70/H30), 60% (D60/H40), and 50% (D50/H50) molar content of protonated, charged D monomer units and PLL in the medium was added to the wells to obtain final concentrations of 1, 10, 30, 100 and 500 µg/mL of the copolymer. Untreated cells served as a positive control. After 72 hours of culture, the cells were subjected to the PrestoBlue® Cell Viability Reagent (Thermo Fisher Scientific, Prague, Czech Republic) in accordance with the manufacturer's protocol.

Fluorescence was measured at an excitation/emission wavelengths of 530/25 nm and 590/35 nm using the Synergy Microplate Reader (Agilent, Fisher Scientific, France). Results were normalized to untreated control values set as 100%. Three independent experiments with three parallels were performed, and data were expressed as a percentage relative to the positive control (100%).

Immunofluorescence staining of the nucleus and von Willebrand factor

The cells were seeded into 24-well glass-bottom plates (20,000 cells per well) and allowed to adhere for 24 hours. The cells were then treated with the D/H polycations containing 60% (D60/H40) and 80% (D80/H20) of charged D monomer units, and PLL (used here as a highly cationic reference control) at final concentrations of 10, 30, and 100 µg/mL for 72 hours. Untreated cells served as a positive control. The cells were then fixed with 4% paraformaldehyde at room temperature (RT) for 15 minutes, washed twice with phosphate-buffered saline (PBS) (Sigma-Aldrich, Prague, Czech Republic). Then, the samples were incubated with 1% albumin + 0.1% TritonX-100 in PBS (20 min, RT), washed with PBS, and incubated with 1% Tween in PBS for 20 min at RT. The cells were incubated with anti-von Willebrand factor antibody (F3520, dilution 1:200, F3520, Merck, Germany) overnight at 4 - 8 °C, subsequently washed twice with PBS, and incubated with Goat anti-Rabbit IgG (H+L) secondary antibody, Alexa Fluor™ 546 (A-11010, dilution 1:400, Thermo Fisher Scientific, Inc.) with Hoechst 33258 (5 µg/mL in PBS; Sigma-Aldrich) at RT in darkness for 60 min. After washing twice with PBS, microphotographs were captured using an epifluorescence microscope (Olympus IX 71 equipped with DP 80 digital camera, Olympus, Japan) with a 10× objective. Cell numbers were counted, and cell densities were calculated using ImageJ FIJI software (v. 1.54f) and Cellpose software v3.0.11.

RESULTS

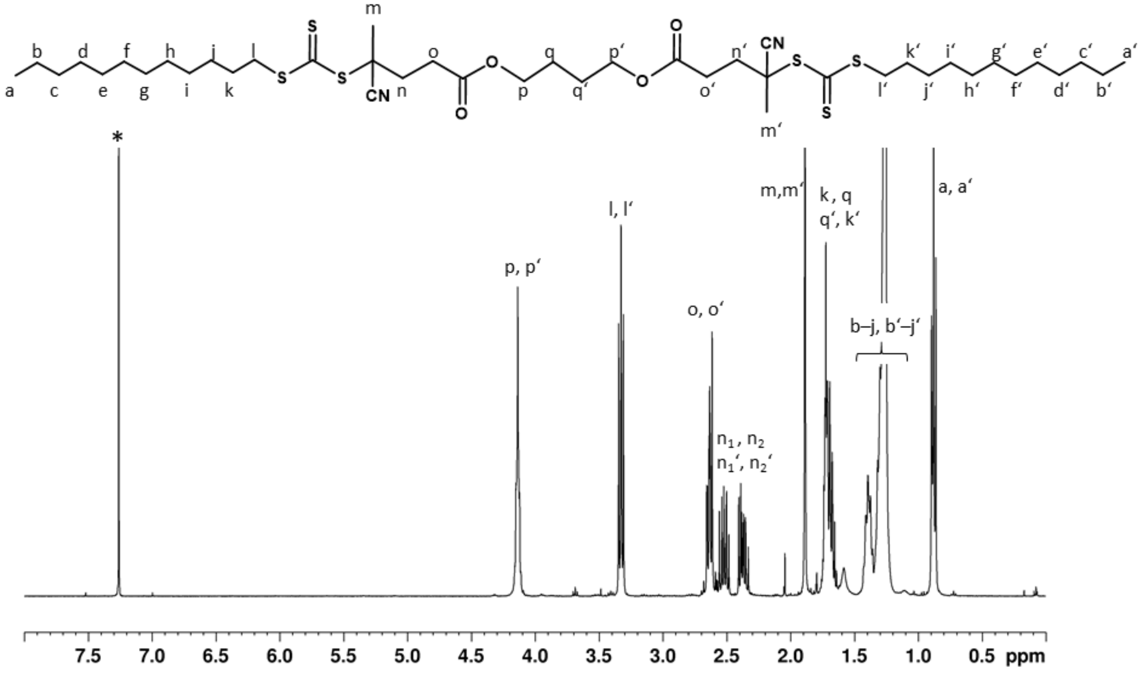

Figure S1. ^{1}H NMR spectrum of the bifunctional CTA in $CDCl_3$. “*” – solvent peak. Signals assignment (400 MHz, $CDCl_3$): δ= 4.20–4.10 (m, 4 H), 3.34 (t, 4 H, J = 7.4 Hz), 2.68–2.61 (m, 4 H), 2.59–2.48 (m, 2 H), 2.43–2.32 (m, 2 H), 1.90 (s, 6 H), 1.78–1.64 (m, 8 H), 1.46–1.20 (m, 36 H), 0.89 (t, 6 H, J = 6.9 Hz) ppm.

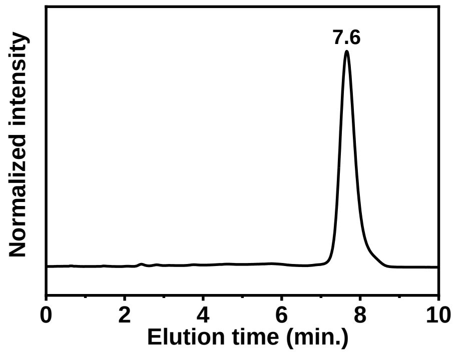

Figure S2. HPLC curve of the bifunctional CTA. CTA was analyzed using a high-performance liquid chromatography Shimadzu system equipped with an SPDM20A photodiode array detector (Shimadzu, Japan), and a column Cosmosil 5C4-AR-300, 150 × 4.6 mm. Gradient elution was performed with 70–95 % of acetonitrile (with 0.1% TFA) for 10 min at a flow rate of 3.0 mL/min.

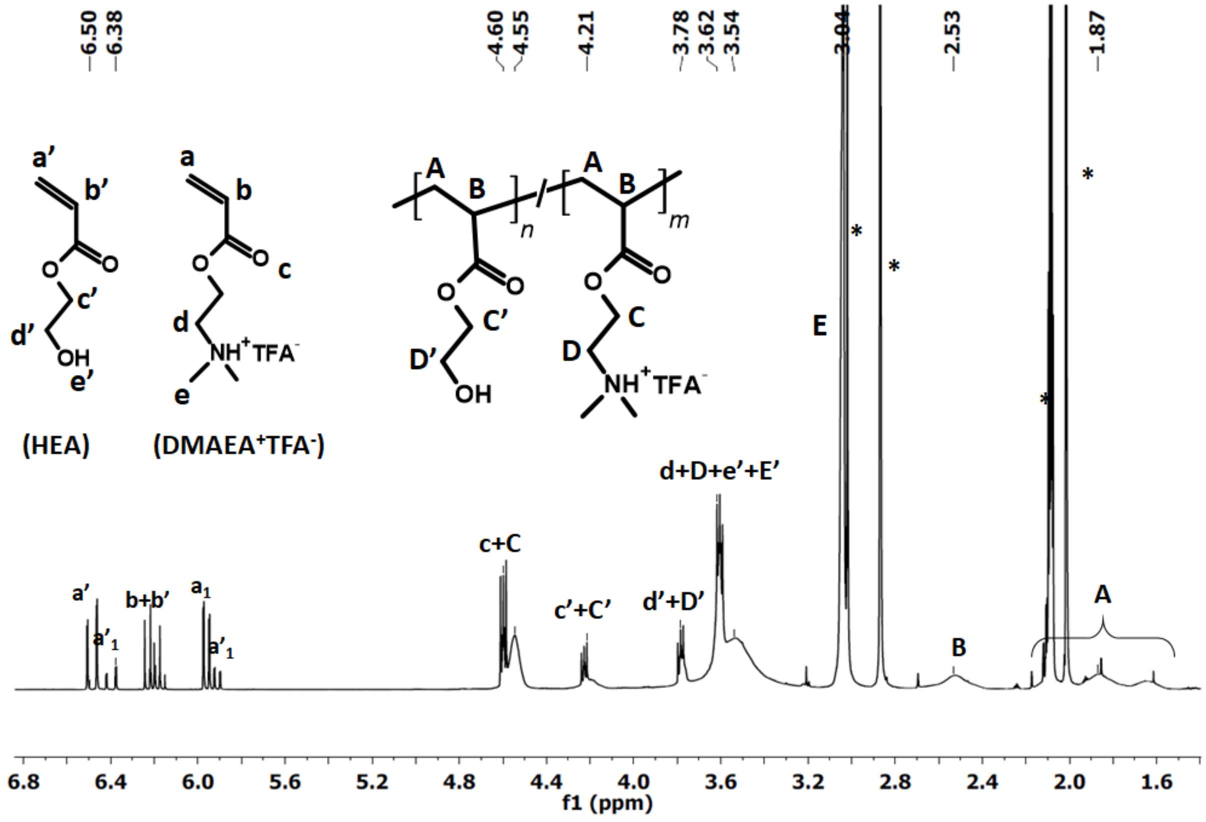

Figure S3. ^{1}H NMR spectrum of the P(DMAEA^{+}TFA^{-}-*co*-HEA) polymerization solution in acetone-d_6 at 75% conversion. The polymerization feed contained 80 mol % DMAEA^{+}TFA^{-} and 20 mol % HEA (i.e., targeting D80/H20 copolymer product). “*” – solvent peak.

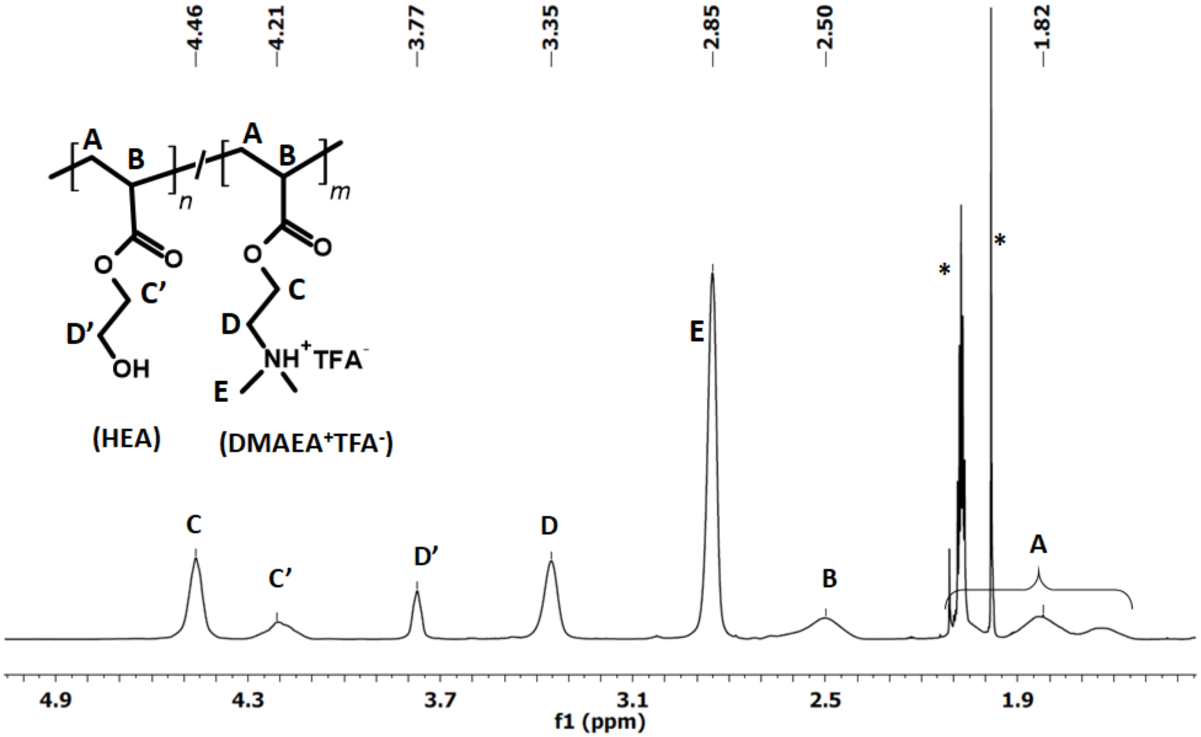

Figure S4. ^{1}H NMR spectrum of D70/H30 copolymer in acetone-d_6. The polymerization feed contained 70 mol% DMAEA$^+$TFA$^-$ and 30 mol% HEA. "*" – solvent peak.

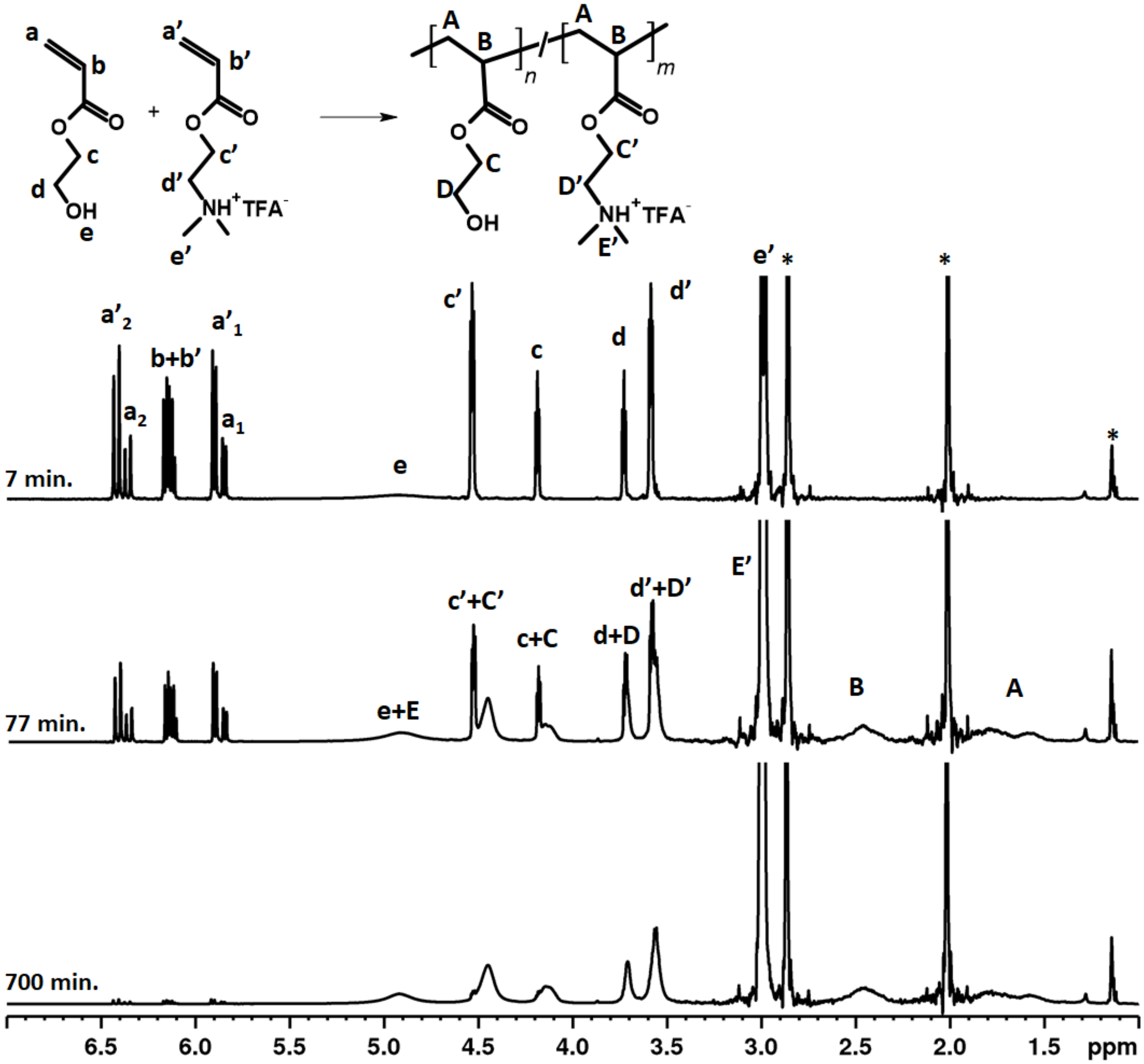

Figure S5. ^{1}H NMR spectra of the DMAEA$^+$TFA$^-$/HEA polymerization solution in *tert*-butanol-d_{10}/DMA (70/30 (v/v)) after 7, 77, and 700 min of polymerization under the same instrumental conditions. The polymerization feed contained 65 mol% DMAEA$^+$TFA$^-$ and 35 mol% HEA (D65/H35). "*" – solvent peak

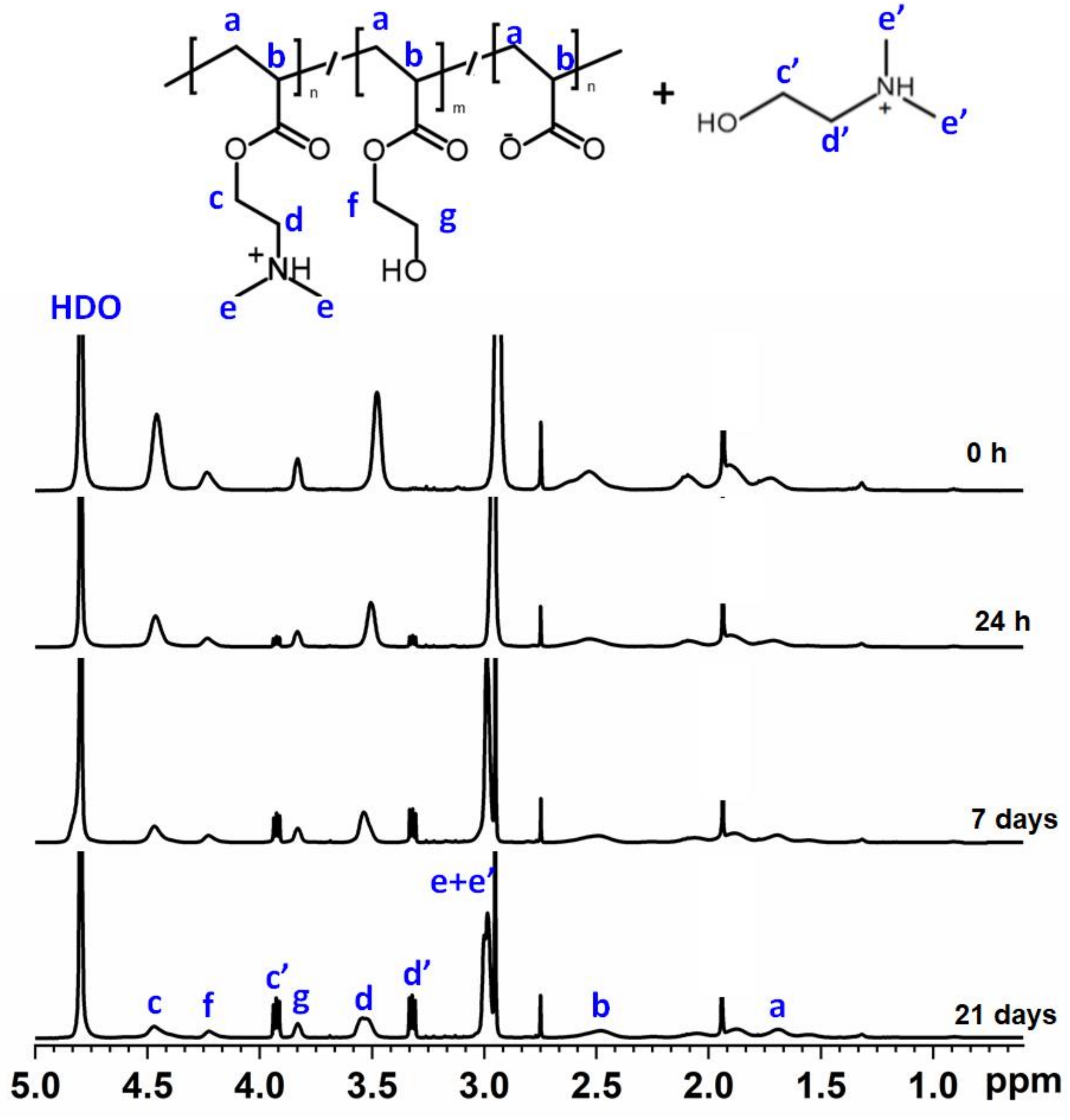

Figure S6. Representative ^{1}H NMR spectra of the D70/H30 copolymer incubated in saline/phosphate-buffered D_2O (0.065 M) at pH 7.4. Room temperature.

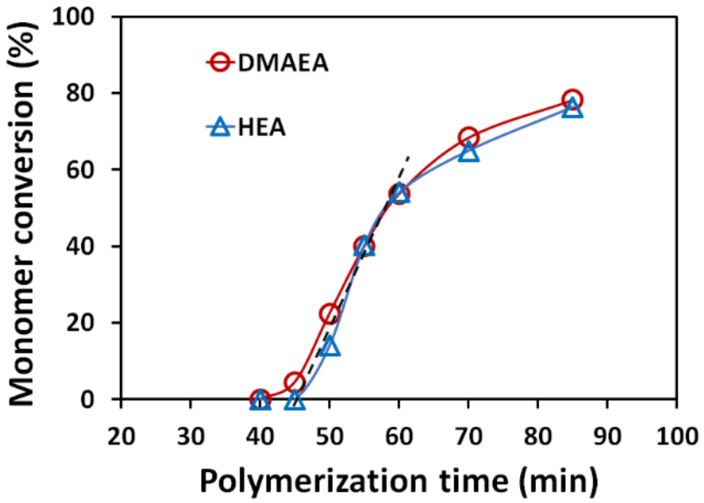

Figure S7. Monomer conversion versus polymerization time for the D85/H15 copolymer prepared by bench-scale RAFT polymerization. Conditions: [M]/[CTA] = 500, [CTA]/[V70] = 5, [M] = 2 M in *tert*-butanol/DMA 70/30 vol./vol. solvent mixtures, 40 °C. (^{1}H NMR spectroscopy)

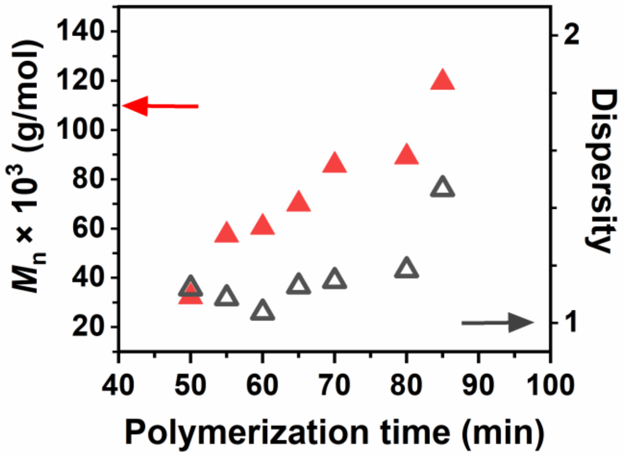

Figure S8. Dependence of M_n (red triangle) and $Đ$ (empty gray triangle) on the polymerization time for $DMAEA^{+}TFA^{-}$/HEA copolymerization of 80% $DMAEA^{+}TFA^{-}$ and 20% HEA in the polymerization feed (D80/H20)

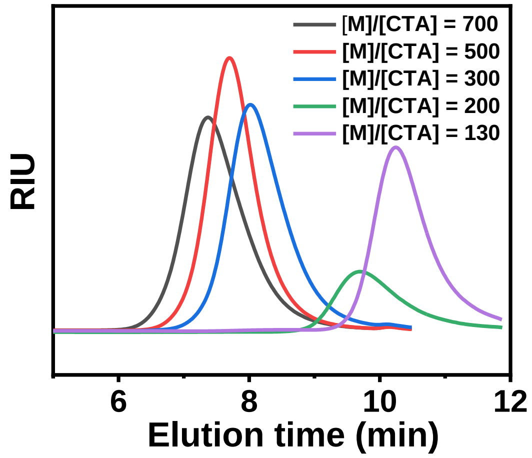

Figure S9. SEC traces of D80/H20 copolymers with increasing [M]/[CTA] ratio. Polymerizations conditions: [M]/[CTA] = 700; 500; 300; 200; 130, [CTA]/[V70] = 5, [M] = 2 M in *tert*-butanol/DMA 70/30 vol./vol. solvent mixtures, 40 °C.

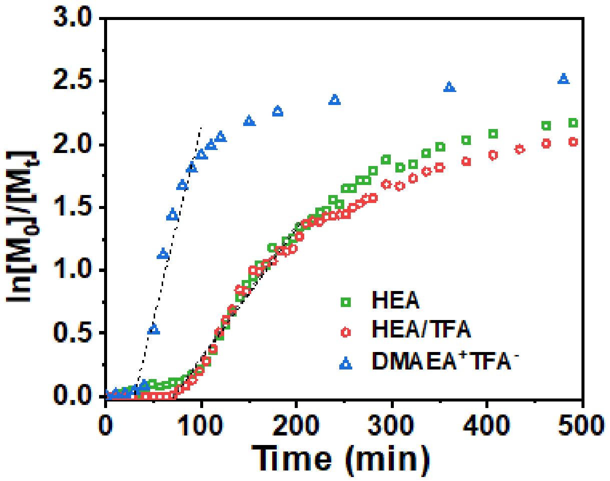

Figure S10. Pseudo-first-order kinetic plots of HEA, HEA/TFA (0.1 mol% relative to HEA), and DMAEA$^+$TFA$^-$ homopolymerizations, showing ln([M_0]/[M_t]) as a function of time. Linear fits were applied to the regions 70–200 min for HEA and HEA/TFA, and 28–98 min for DMAEA$^+$TFA$^-$. All polymerizations were performed at [M]/[CTA] = 450, [CTA]/[V70] = 5, [M] = 2 M in *tert*-butanol-d_{10}/DMA (30 vol %).

The apparent first-order rate constants (k_{app}) were determined as 0.0106, 0.01059, and 0.0335 min^{-1} for HEA, HEA/TFA, and DMAEA$^+$TFA$^-$, respectively, evidencing the lower rate of HEA homopolymerization compared to DMAEA$^+$TFA$^-$

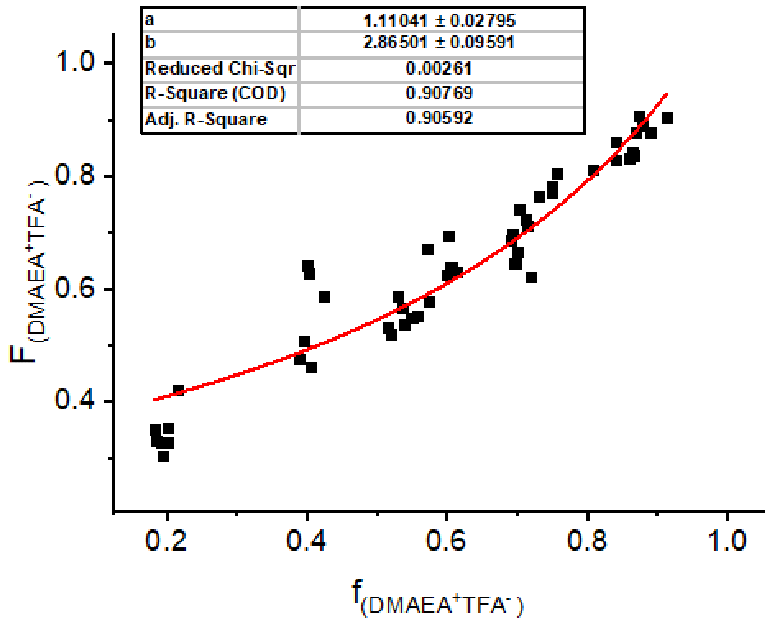

Figure S11. Experimental copolymer composition in DMAEA^{+}TFA^{-} units, $F_{(\mathrm{DMAEA+TFA-})}$, as a function of the DMAEA^{+}TFA^{-} feed fraction, $f_{(\mathrm{DMAEA+TFA-})}$, fitted to the Mayo–Lewis model. The obtained reactivity ratios are $r_{(\mathrm{DMAEA})}$ = 1.114 ± 0.027 and $r_{(\mathrm{HEA})}$ = 2.865 ± 0.096, indicating nearly statistical propagation of DMAEA^{+}TFA^{-} radicals and moderate self-propagation tendency of HEA radicals.

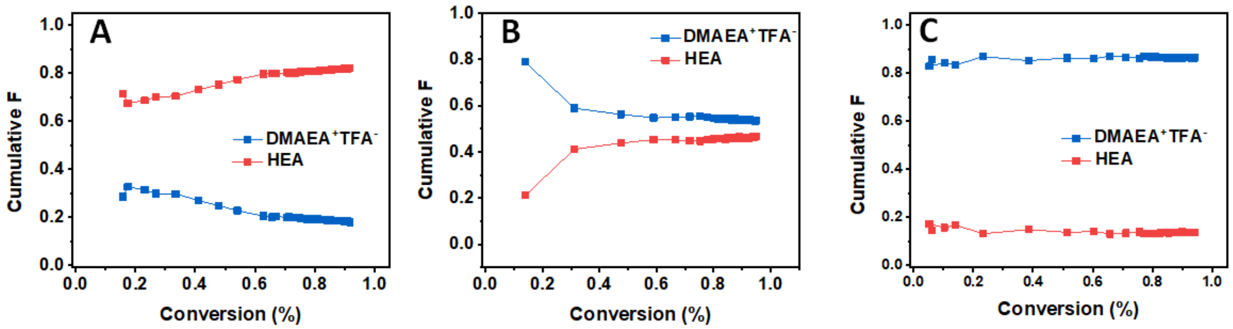

Figure S12. Experimental cumulative copolymer compositions, F, for DMAEA^{+}TFA^{-}/HEA (D/H) copolymers for three representative feeds: (A) HEA-rich feed (D15/H85); (B) equimolar feed (D50/H50); and (C) HEA-lean feed (D85/H15). The numbers after the monomer abbreviations denotes the molar % content of monomers in the feed.

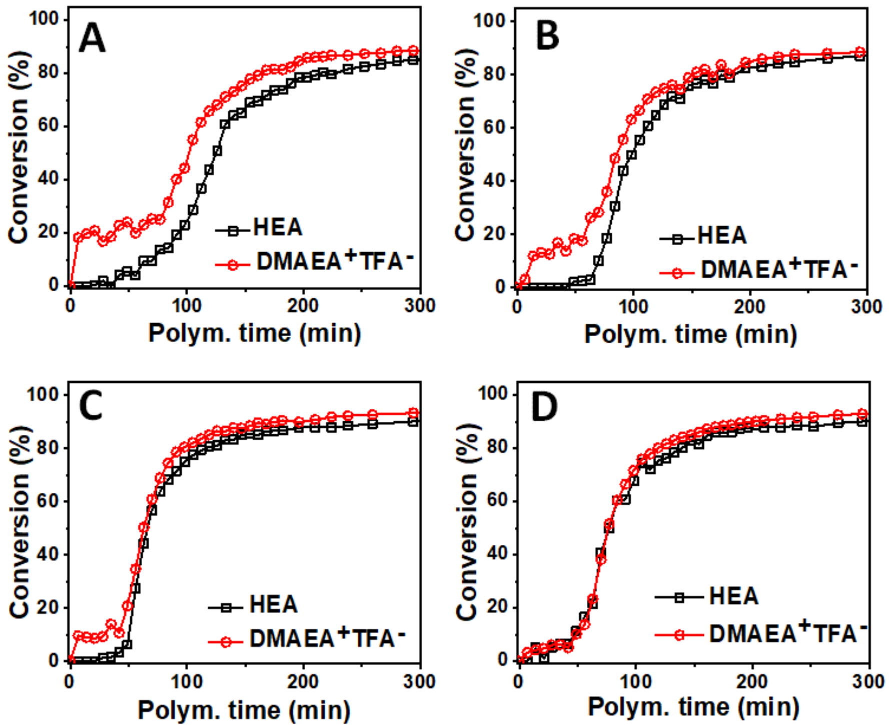

Figure S13. ^{1}H NMR kinetic curves of DMAEA$^+$TFA$^-$/HEA copolymerizations with different monomer contents in the polymerization feed: (A) D15/H85; (B) D35/H65; (C) D50/H50; (D) D85/H15. In the D/H notation, the numbers denote the molar percentage of monomers in the polymerization feed. All polymerizations were performed at [M]/[CTA] = 450, [CTA]/[V70] = 5, [M] = 2 M in *tert*-butanol-d_{10}/DMA (70/30 (v/v)).

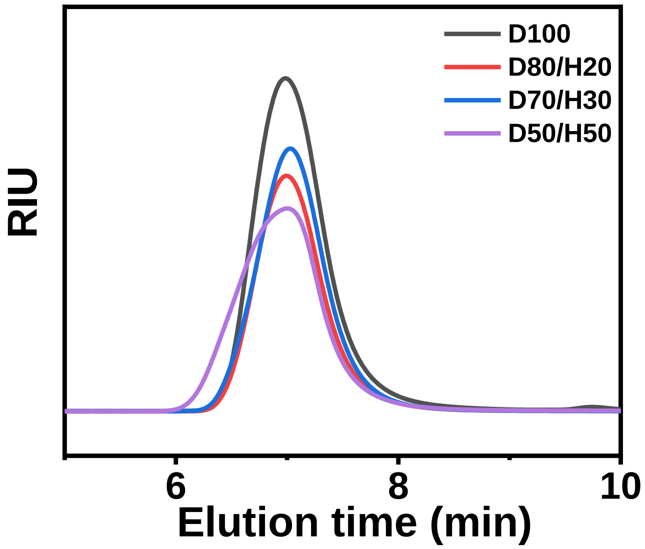

Figure S14. SEC traces of DMAEA$^+$TFA$^-$/HEA copolymers with various comonomer ratios (polymerization conversions up to 85%). Polymerizations conditions: [M]/[CTA] = 500, [CTA]/[V70] = 5, [M] = 2 M in *tert*-butanol/DMA 70/30 (vol/vol) solvent mixtures, 40 °C.

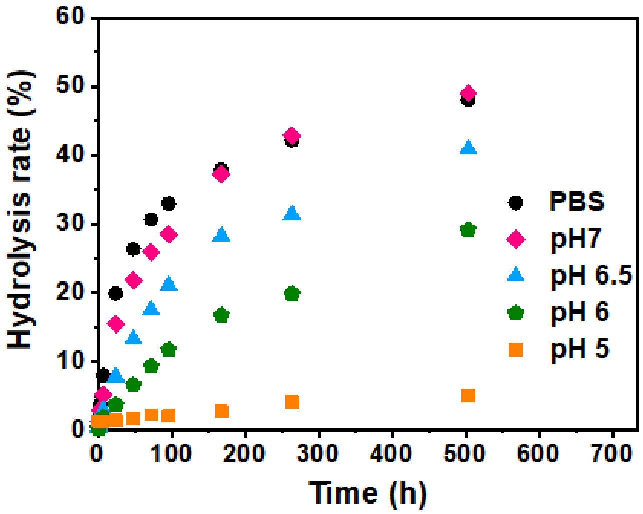

Figure S15. Effect of pH on the hydrolysis of 0.5% wt. solutions of PDMAEA$^+$TFA$^-$ homopolymer. Buffers: 0.063 M saline/phosphate-buffered D_2O of pH 5, 6, 6.5, 7, and 7.4; room temperature.

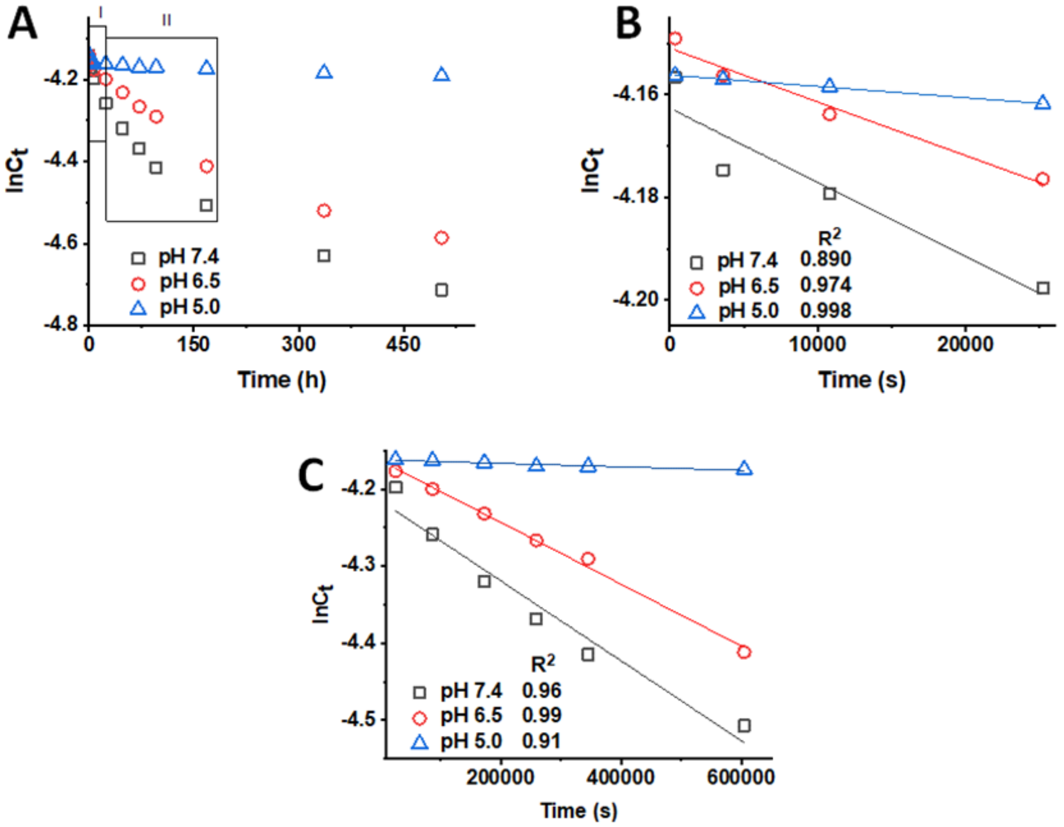

Figure S16. Effect of pH on the hydrolysis of D70/H30. (A) Semilogarithmic plots of the $DMAEA^+TFA^-$ concentration versus time. Regions I and II showing linear dependence are indicated by boxes. (B) Linear fits of semilogarithmic plots of the $DMAEA^+TFA^-$ concentration versus time during 24 h of hydrolysis (region I). (C) Linear fits of semilogarithmic plots of the $DMAEA^+TFA^-$ concentration versus time during 24–168 h of hydrolysis (region II).

Table S1. Initial rate constants k_I and k_{II} of the hydrolysis of D70/H30 copolymer at different pH values, calculated for regions I and II, are presented in **Figure S13**

pH	C_0[a]	k_I	STD	conversion[b]	k_{II}	STD	conversion[c]
	(M)	($\times10^{-6}$ s^{-1})	($\times10^{-7}$ s^{-1})	(%)	($\times10^{-7}$ s^{-1})	($\times10^{-8}$ s^{-1})	(%)
7.4		1.44	3.6	11.1	5.20	5.2	30.6
6.5	0.0159	1.05	1.2	5.7	4.01	1.4	23.7
5.0		0.22	0.1	2.1	0.22	0.4	3.2

[a] ..initial $DMAEA^+TFA^-$ concentration, [b] .. conversion of $DMAEA^+TFA^-$ at the end of region I; [c]..conversion of $DMAEA^+TFA^-$ at the end of region II

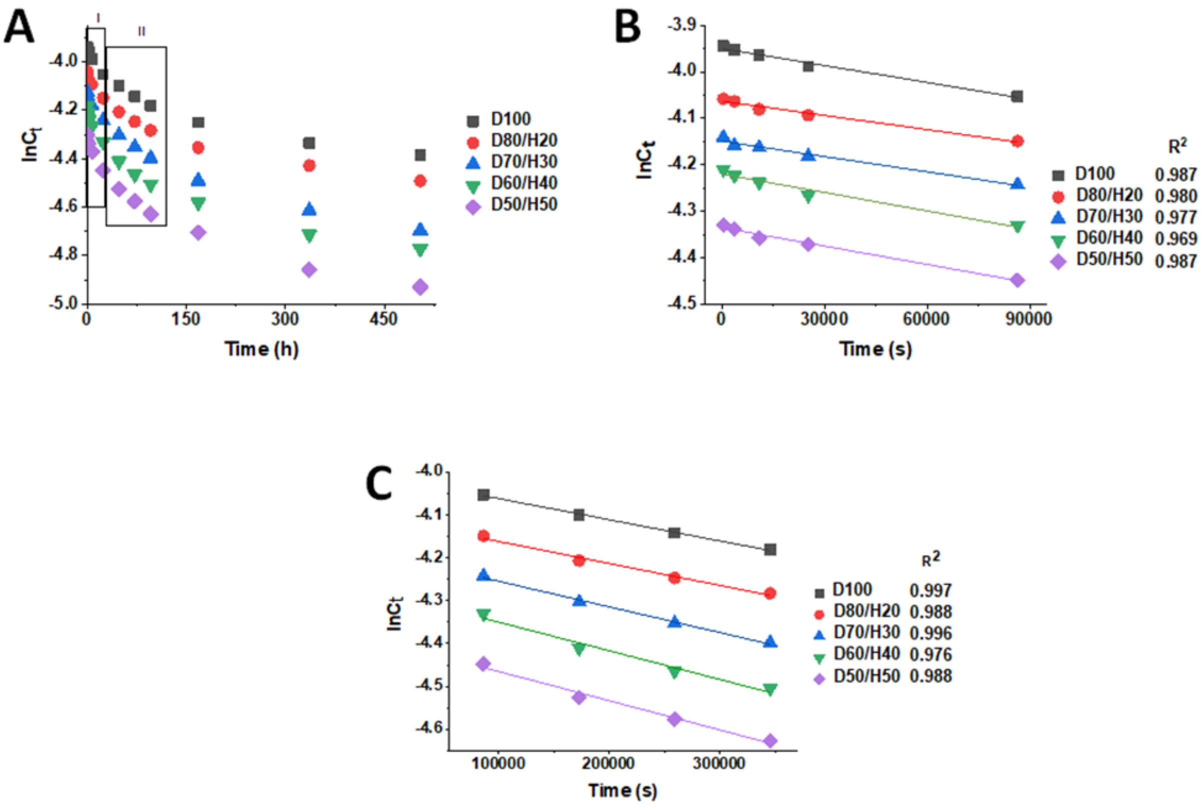

Figure S17. Effect of the HEA content on the hydrolysis of D/H copolymers at pH 7.4. (A) Semilogarithmic plots of the DMAEA$^+$TFA$^-$ concentration versus time. Regions I and II showing linear dependence are indicated by boxes. (B) Linear fits of semilogarithmic plots of the DMAEA+TFA$^-$ concentration versus time during 24 h of hydrolysis (region I). (C) Linear fits of semilogarithmic plots of the DMAEA$^+$TFA$^-$ concentration versus time during 24–168 h of hydrolysis (region II). Polycations tested included D/H copolymers with 100% (D100), 80% (D80/H20), 70% (D70/H30), 60% (D60/H40), and 50% (D50/H50) molar content of protonated, charged D monomer units.

Table S2. Initial rate constants k_I and k_{II} of the hydrolysis of D/H copolymers with varying charge content at pH 7.4, calculated for regions I and II, are presented in **Figure S17**.

Sample	C_0[a]	k_I	STD	conversion[b]	k_{II}	STD	conversion[c]
	(M)	($\times 10^{-6}$ s^{-1})	($\times 10^{-7}$ s^{-1})	(%)	($\times 10^{-7}$ s^{-1})	($\times 10^{-8}$ s^{-1})	(%)
100	0.0194	1.22	8.1	10.6	4.94	1.8	21.4
D80/20	0.0175	1.02	8.5	10.2	5.12	4.0	21.5
D70/30	0.0162	1.10	9.7	11.1	5.98	2.8	23.9
D60/40	0.0152	1.32	13.6	13.5	6.68	7.3	27.4
D50/50	0.0136	1.32	8.2	13.7	6.82	5.3	27.9

[a] ..initial $DMAEA^{+}TFA^{-}$ concentration, [b] .. conversion of $DMAEA^{+}TFA^{-}$ at the end of region I; [c]..conversion of $DMAEA^{+}TFA^{-}$ at the end of region II

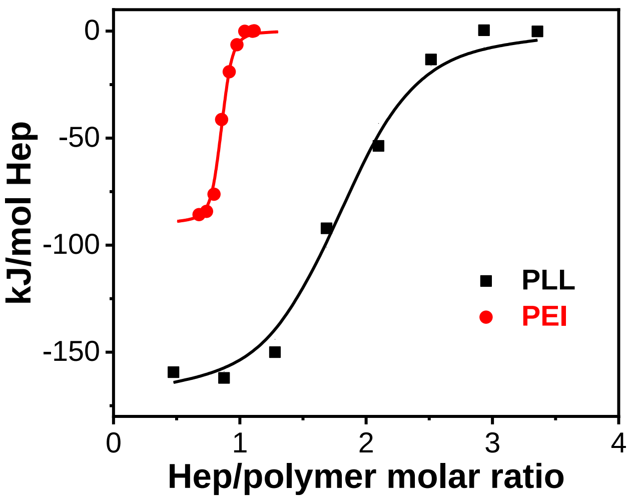

Figure S18. Titration isotherms of PLL and PEI solutions to a heparin (Hep) solution in saline/phosphate buffer at pH 6.5 (isothermal titration calorimetry).

Table S3. Thermodynamic parameters of the interaction of heparin with PLL and PEI in saline/phosphate buffer at pH 6.5 (ITC determination).

**Sample**	***n***	K_A $\cdot 10^6$ M^{-1}	**Δ*H*** kJ/mol	**Δ*S*** J/mol·K	**Δ*G*** kJ/mol
PLL	2.0	0.79	-169.8	-456	-33.6
PEI	~1	3.50	-90	-176	-37.3

n - binding stoichiometry (a number of heparin molecules bound to the copolymer molecule), K_A - binding affinity constant, Δ*H* – binding enthalpy, Δ*S* – binding entropy, Δ*G* - Gibbs free energy of binding

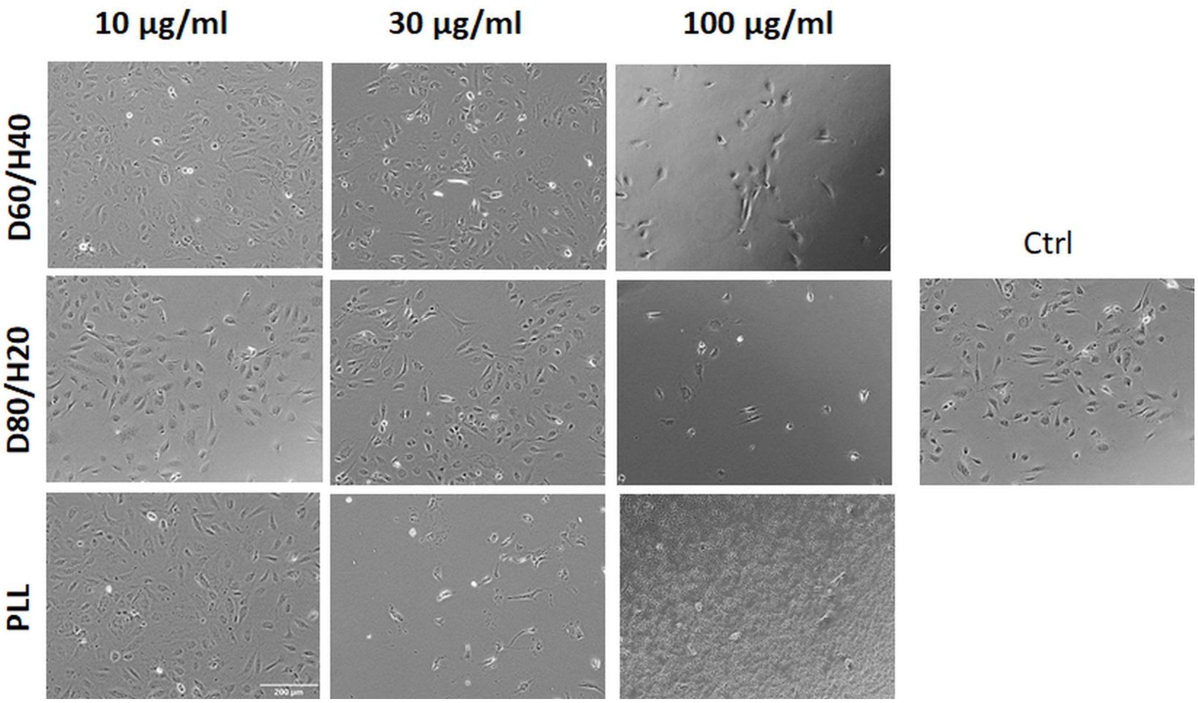

Figure S19. The morphology and spreading of HUVECs cultured for 24 hours in the presence of polycations. Images of native cells were taken using an epifluorescence microscope (Olympus IX71) equipped with a DP 80 digital camera (objective × 10, scale bar = 200 µm). Polycations tested included D/H copolymers with 80% (D80/H20) and 60% (D60/H40) molar content of protonated, charged D monomer units. Poly(L-lysine) (PLL) was used as a negative control, and untreated cells served as a positive control.

Table S4. Statistical analysis: Effect of the charge content in D/H copolymers on HUVEC cell index at fixed polycation concentrations (500−1 μg/ml). One-way ANOVA with Student-Newman-Keuls multiple comparison test. D/H copolymers with 100% (D100), 80% (D80/H20), 70% (D70/H30), 60% (D60/H40), and 50% (D50/H50) molar contents of $DMAEA^{+}TFA^{-}$ monomer units. Polylysine (PLL).

500 μg/ml polycation concentration

	D100-500	D80/H20-500	D70/H30-500	D60/H40-500	D50/50-500	PLL-500
**D100-500**	-	n.s.	n.s.	n.s.	0.001	n.s.
**D80/H20-500**	n.s.	-	n.s.	n.s.	0.001	n.s.
**D70/H30-500**	n.s.	n.s.	-	n.s.	0.001	n.s.
**D60/H40-500**	n.s.	n.s.	n.s.	-	0.001	n.s.
**D50/50-500**	0.001	0.001	0.001	0.001	-	0.001
**PLL-500**	n.s.	n.s.	n.s.	n.s.	0.001	-

100 μg/ml polycation concentration

	D100-100	D80/H20-100	D70/H30-100	D60/H40-100	D50/50-100	PLL-100
**D100-100**	-	n.s.	n.s.	n.s.	0.001	n.s.
**D80/H20-100**	n.s.	-	n.s.	n.s.	0.001	n.s.
**D70/H30-100**	n.s.	n.s.	-	n.s.	0.001	n.s.
**D60/H40-100**	n.s.	n.s.	n.s.	-	0.001	n.s.
**D50/50-100**	0.001	0.001	0.001	0.001	-	0.001
**PLL-100**	n.s.	n.s.	n.s.	n.s.	0.001	-

30 μg/ml polycation concentration

	D100-30	D80/H20-30	D70/H30-30	D60/H40-30	D50/50-30	PLL-30
**D100-30**	-	n.s.	n.s.	0.05	n.s.	0.001
**D80/H20-30**	n.s.	-	n.s.	n.s.	n.s.	0.001
**D70/H30-30**	n.s.	n.s.	-	n.s.	n.s.	0.001
**D60/H40-30**	0.05	n.s.	n.s.	-	n.s.	0.001
**D50/50-30**	n.s.	n.s.	n.s.	n.s.	-	0.001
**PLL-30**	0.001	0.001	0.001	0.001	0.001	-

No statistically significant differences among the samples were observed at polycation concentrations of 10 and 1 μg/mL.

Table S5. Statistical analysis. Effect of the polycation concentration on HUVEC cell index at a fixed charge content of D/H copolymers. One-way ANOVA with Student-Newman-Keuls multiple comparison test. D/H copolymers with 100% (D100), 80% (D80/H20), 70% (D70/H30), 60% (D60/H40), and 50% (D50/H50) molar contents of DMAEA^{+}TFA^{-} monomer units. Polylysine (PLL). Polycation concentrations: 500, 100, 30, 10, and 1 μg/ml.

D100 homopolymer

	D100-500	D100-100	D100-30	D100-10	D100-1	Ctrl
**D100-500**	-	n.s.	0.001	0.001	0.001	0.001
**D100-100**	n.s.	-	0.001	0.001	0.001	0.001
**D100-30**	0.001	0.001	-	0.001	0.001	0.001
**D100-10**	0.001	0.001	0.001	-	n.s.	n.s.
**D100-1**	0.001	0.001	0.001	n.s.	-	n.s.
**Ctrl**	0.001	0.001	0.001	n.s.	n.s.	-

D80/H20 copolymer

	D80/H20-500	D80/H20-100	D80/H20-30	D80/H20-10	D80/H20-1	Ctrl
**D80/H20-500**	-	n.s.	0.001	0.001	0.001	0.001
**D80/H20-100**	n.s.	-	0.001	0.001	0.001	0.001
**D80/H20-30**	0.001	0.001	-	0.001	0.001	0.001
**D80/H20-10**	0.001	0.001	0.001	-	n.s.	n.s.
**D80/H20-1**	0.001	0.001	0.001	n.s.	-	n.s.
**Ctrl**	0.001	0.001	0.001	n.s.	n.s.	-

D70/H30 copolymer

	D70/H30-500	D70/H30-100	D70/H30-30	D70/H30-10	D70/H30-1	Ctrl
**D70/H30-500**	-	n.s.	0.001	0.001	0.001	0.001
**D70/H30-100**	n.s.	-	0.001	0.001	0.001	0.001
**D70/H30-30**	0.001	0.001	-	0.001	0.001	0.001
**D70/H30-10**	0.001	0.001	0.001	-	n.s.	n.s.
**D70/H30-1**	0.001	0.001	0.001	n.s.	-	n.s.
**Ctrl**	0.001	0.001	0.001	n.s.	n.s.	-

D60/H40 copolymer

	D60/H40-500	D60/H40-100	D60/H40-30	D60/H40-10	D60/H40-1	Ctrl
**D60/H40-500**	-	n.s.	0.001	0.001	0.001	0.001
**D60/H40-100**	n.s.	-	0.001	0.001	0.001	0.001
**D60/H40-30**	0.001	0.001	-	0.001	0.01	0.01
**D60/H40-10**	0.001	0.001	0.001	-	n.s.	n.s.
**D60/H40-1**	0.001	0.001	0.01	n.s.	-	n.s.
**Ctrl**	0.001	0.001	0.01	n.s.	n.s.	-

Table continues on the following page

Table S5 – Continued

D50/H50 copolymer

	D50/50-500	D50/50-100	D50/50-30	D50/50-10	D50/50-1	Ctrl
**D50/50-500**	-	n.s.	0.001	n.s.	n.s.	n.s.
**D50/50-100**	n.s.	-	0.05	n.s.	n.s.	n.s.
**D50/50-30**	0.001	0.05	-	0.001	0.001	0.001
**D50/50-10**	n.s.	n.s.	0.001	-	n.s.	n.s.
**D50/50-1**	n.s.	n.s.	0.001	n.s.	-	n.s.
**Ctrl**	n.s.	n.s.	0.001	n.s.	n.s.	-

Polylysin (PLL) homopolymer

	PLL-500	PLL-100	PLL-30	PLL-10	PLL-1	Ctrl
**PLL-500**	-	n.s.	n.s.	0.001	0.001	0.001
**PLL-100**	n.s.	-	n.s.	0.001	0.001	0.001
**PLL-30**	n.s.	n.s.	-	0.001	0.001	0.001
**PLL-10**	0.001	0.001	0.001	-	n.s.	n.s.
**PLL-1**	0.001	0.001	0.001	n.s.	-	n.s.
**Ctrl**	0.001	0.001	0.001	n.s.	n.s.	-

Table S6. Statistical analysis: Effect of the charge content in D/H copolymers on cell viability at fixed polycation concentrations (500–1 μg/ml). One-way ANOVA with Student-Newman-Keuls multiple comparison test. D/H copolymers with 100% (D100), 80% (D80/H20), 70% (D70/H30), 60% (D60/H40), and 50% (D50/H50) molar contents of DMAEA^{+}TFA^{-} monomer units. Polylysine (PLL).

500 μg/ml polycation concentration

	D100-500	D80/H20-500	D70/H30-500	D60/H40-500	D50/50-500	PLL-500
**D100-500**	-	n.s.	n.s.	n.s.	0.001	n.s.
**D80/H20-500**	n.s.	-	n.s.	n.s.	0.001	n.s.
**D70/H30-500**	n.s.	n.s.	-	n.s.	0.001	n.s.
**D60/H40-500**	n.s.	n.s.	n.s.	-	0.001	n.s.
**D50/50-500**	0.001	0.001	0.001	0.001	-	0.001
**PLL-500**	n.s.	n.s.	n.s.	n.s.	0.001	-

100 μg/ml polycation concentration

	D100-100	D80/H20-100	D70/H30-100	D60/H40-100	D50/50-100	PLL-100
**D100-100**	-	n.s.	0.001	0.001	0.001	0.01
**D80/H20-100**	n.s.	-	0.001	0.001	0.001	0.01
**D70/H30-100**	0.001	0.001	-	0.001	0.001	0.001
**D60/H40-100**	0.001	0.001	0.001	-	0.001	0.001
**D50/50-100**	0.001	0.001	0.001	0.001	-	0.001
**PLL-100**	0.01	0.01	0.001	0.001	0.001	-

30 μg/ml polycation concentration

	D100-30	D80/H20-30	D70/H30-30	D60/H40-30	D50/50-30	PLL-30
**D100-30**	-	0.01	0.001	0.001	0.001	0.001
**D80/H20-30**	0.01	-	n.s.	n.s.	n.s.	0.001
**D70/H30-30**	0.001	n.s.	-	n.s.	n.s.	0.001
**D60/H40-30**	0.001	n.s.	n.s.	-	n.s.	0.001
**D50/50-30**	0.001	n.s.	n.s.	n.s.	-	0.001
**PLL-30**	0.001	0.001	0.001	0.001	0.001	-

No statistically significant differences among the samples were observed at polycation concentrations of 10 and 1 μg/mL.

Table S7. Statistical analysis. Effect of the polycation concentration on cell viability at a fixed charge content of D/H copolymers. One-way ANOVA with Student-Newman-Keuls multiple comparison test. D/H copolymers with 100% (D100), 80% (D80/H20), 70% (D70/H30), 60% (D60/H40), and 50% (D50/H50) molar contents of $DMAEA^{+}TFA^{-}$ monomer units. Polylysine (PLL). Polycation concentrations: 500, 100, 30, 10, and 1 μg/ml

D100 homopolymer

	D100-500	D100-100	D100-30	D100-10	D100-1	Ctrl
**D100-500**	-	n.s.	0.001	0.001	0.001	0.001
**D100-100**	n.s.	-	0.001	0.001	0.001	0.001
**D100-30**	0.001	0.001	-	0.001	0.001	0.001
**D100-10**	0.001	0.001	0.001	-	n.s.	n.s.
**D100-1**	0.001	0.001	0.001	n.s.	-	n.s.
**Ctrl**	0.001	0.001	0.001	n.s.	n.s.	-

D80/H20 copolymer

	D80/H20-500	D80/H20-100	D80/H20-30	D80/H20-10	D80/H20-1	Ctrl
**D80/H20-500**	-	n.s.	0.001	0.001	0.001	0.001
**D80/H20-100**	n.s.	-	0.001	0.001	0.001	0.001
**D80/H20-30**	0.001	0.001	-	0.001	0.001	0.001
**D80/H20-10**	0.001	0.001	0.001	-	n.s.	n.s.
**D80/H20-1**	0.001	0.001	0.001	n.s.	-	n.s.
**Ctrl**	0.001	0.001	0.001	n.s.	n.s.	-

D70/H30 copolymer

	D70/H30-500	D70/H30-100	D70/H30-30	D70/H30-10	D70/H30-1	Ctrl
**D70/H30-500**	-	0.001	0.001	0.001	0.001	0.001
**D70/H30-100**	0.001	-	0.001	0.001	0.001	0.001
**D70/H30-30**	0.001	0.001	-	0.001	0.001	0.001
**D70/H30-10**	0.001	0.001	0.001	-	n.s.	n.s.
**D70/H30-1**	0.001	0.001	0.001	n.s.	-	n.s.
**Ctrl**	0.001	0.001	0.001	n.s.	n.s.	-

D60/H40 copolymer

	D60/H40-500	D60/H40-100	D60/H40-30	D60/H40-10	D60/H40-1	Ctrl
**D60/H40-500**	-	0.001	0.001	0.001	0.001	0.001
**D60/H40-100**	0.001	-	0.001	0.001	0.001	0.001
**D60/H40-30**	0.001	0.001	-	n.s.	n.s.	0.05
**D60/H40-10**	0.001	0.001	n.s.	-	n.s.	n.s.
**D60/H40-1**	0.001	0.001	n.s.	n.s.	-	n.s.
**Ctrl**	0.001	0.001	0.05	n.s.	n.s.	-

Table continues on the following page

Table S7- Continued

D50/H50 copolymer

	D50/50-500	D50/50-100	D50/50-30	D50/50-10	D50/50-1	Ctrl
**D50/50-500**	-	n.s.	n.s.	n.s.	n.s.	n.s.
**D50/50-100**	n.s.	-	n.s.	n.s.	n.s.	n.s.
**D50/50-30**	n.s.	n.s.	-	n.s.	n.s.	n.s.
**D50/50-10**	n.s.	n.s.	n.s.	-	n.s.	n.s.
**D50/50-1**	n.s.	n.s.	n.s.	n.s.	-	n.s.
**Ctrl**	n.s.	n.s.	n.s.	n.s.	n.s.	-

Polylysin (PLL) homopolymer, negative control

	PLL-500	PLL-100	PLL-30	PLL-10	PLL-1	Ctrl
**PLL-500**	-	n.s.	n.s.	0.001	0.001	0.001
**PLL-100**	n.s.	-	n.s.	0.001	0.001	0.001
**PLL-30**	n.s.	n.s.	-	0.001	0.001	0.001
**PLL-10**	0.001	0.001	0.001	-	n.s.	n.s.
**PLL-1**	0.001	0.001	0.001	n.s.	-	n.s.
**Ctrl**	0.001	0.001	0.001	n.s.	n.s.	-
